# Systematic review and critique of circulating miRNAs as biomarkers of stage I-II non-small cell lung cancer

**DOI:** 10.18632/oncotarget.21739

**Published:** 2017-10-11

**Authors:** Francesca Moretti, Paola D’Antona, Emanuele Finardi, Marco Barbetta, Lorenzo Dominioni, Albino Poli, Elisabetta Gini, Douglas M. Noonan, Andrea Imperatori, Nicola Rotolo, Maria Cattoni, Paola Campomenosi

**Affiliations:** ^1^ Department of Diagnostic and Public Health, University of Verona, Verona, Italy; ^2^ Department of Biotechnology and Life Sciences, DBSV, University of Insubria, Varese, Italy; ^3^ Department of Medicine and Surgery, DMS, Center for Thoracic Surgery, University of Insubria, Varese, Italy; ^4^ Scientific and Technological Pole, IRCCS MultiMedica, Milan, Italy; ^5^ The Protein Factory, Centro Interuniversitario di Ricerca in Biotecnologie Proteiche, Politecnico di Milano, ICRM-CNR Milano and University of Insubria, Varese, Italy

**Keywords:** microRNA, biomarkers, circulating, non-small cell lung cancer, stage I-II NSCLC

## Abstract

Selected circulating microRNAs (miRNAs) have been suggested for non-invasive screening of non-small cell lung cancer (NSCLC), however the numerous proposed miRNA signatures are inconsistent.

Aiming to identify miRNAs suitable specifically for stage I-II NSCLC screening in serum/plasma samples, we searched the databases “Pubmed”, “Medline”, “Scopus”, “Embase” and “WOS” and systematically reviewed the publications reporting quantitative data on the efficacy [sensitivity, specificity and/or area under the curve (AUC)] of circulating miRNAs as biomarkers of NSCLC stage I and/or II. The 20 studies fulfilling the search criteria included 1110 NSCLC patients and 1009 controls, and were of medium quality according to Quality Assessment of Diagnostic Accuracy Studies checklist. In these studies, the patient cohorts as well as the control groups were heterogeneous for demographics and clinicopathological characteristics; moreover, numerous pre-analytical and analytical variables likely influenced miRNA determinations, and potential bias of hemolysis was often underestimated. We identified four circulating miRNAs scarcely influenced by hemolysis, each featuring high sensitivity (> 80%) and AUC (> 0.80) as biomarkers of stage I-II NSCLC: miR-223, miR-20a, miR-448 and miR-145; four other miRNAs showed high specificity (> 90%): miR-628-3p, miR-29c, miR-210 and miR-1244. In a model of two-step screening for stage I-II NSCLC using first the above panel of serum miRNAs with high sensitivity and high AUC, and subsequently the panel with high specificity, the estimated overall sensitivity is 91.6% and overall specificity is 93.4%. These and other circulating miRNAs suggested for stage I-II NSCLC screening require validation in multiple independent studies before they can be proposed for clinical application.

## INTRODUCTION

Lung cancer is the most common cause of cancer death worldwide, globally accounting for an estimated 1.5 million deaths in 2012 [1, 2]. In Europe, every year lung cancer causes about 353000 deaths, which represent nearly 20% of total cancer deaths [3]. Approximately 15% of lung cancers are histologically classified as small cell lung cancer, a very aggressive and generally incurable tumor; the remaining 85% are cumulatively classified as Non-Small Cell Lung Cancer (NSCLC). The latter contains two main histological subtypes, adenocarcinoma (AC) and squamous cell carcinoma (SCC), and can often be cured if diagnosed at an early stage [4, 5].

The five-year survival rate of lung cancer is low worldwide (10–15%), mainly because the majority of cases are diagnosed with advanced stage, when treatment is rarely curative. In the NSCLC cases that are diagnosed at an early stage (stage I and II), the five-year survival rate dramatically improves, ranging from 70% to 85% for surgically resected stage I disease [6], lobectomy being the established and most effective therapeutic approach [7]. However, less than one third of NSCLC cases are diagnosed in early stage [8–10] and the methodologies currently available for early diagnosis present several limitations. Chest X-rays have low sensitivity for lung cancer detection, whereas low-dose chest computed tomography (CT) scan has high sensitivity but low specificity [11–14]. The latter is a relevant limitation of CT scan for screening, considering that among individuals at risk for lung cancer (heavy smokers and former smokers) 20–60% of chest CT exams show pulmonary nodules, the vast majority of which are eventually diagnosed as benign after completion of work up [15, 16]. Moreover, in many areas of the world chest CT is a rather expensive and not widely available screening tool [17].

Newer, minimally invasive and effective methods of screening for lung cancer are needed. MicroRNAs (miRNAs) are small non-protein-coding RNA molecules 18–25 nucleotide long that play an important role in eukaryotic gene expression regulation. They have been shown to be dysregulated in human diseases, including cancer [18, 19]. The aberrant expression of specific miRNAs in body fluids from individuals with cancer has suggested their possible application as cancer biomarkers [20–25]. The quantification of selected miRNAs in plasma or serum of high risk individuals has been proposed as a simple and potentially effective screening tool for early detection of NSCLC. Unfortunately, the miRNA signatures identified by numerous published studies of lung cancer patients are largely inconsistent, the reported miRNA profiles being incoherent [23, 26–33]. These studies have been the subject of several reviews and meta-analyses [34–40]. However, these reviews were not focused on circulating miRNAs in cancer stage I and II, potentially amenable to radical cure. Moreover, the accuracy of miRNA quantification in plasma/serum is known to be affected by several methodological variables, including modality of sample preparation, hemolysis, RNA isolation procedures, method of cDNA preparation and method used for miRNA measurement. These factors, that likely contribute to the puzzling inconsistency of the published miRNA profiles of NSCLC, were only partially addressed in the aforementioned reviews.

Here we aimed to review the literature in order to identify circulating miRNAs proven to be valuable and highly accurate for diagnosis of early NSCLC (stage I and II). Further, based on our analysis, we propose two panels of miRNAs for diagnosis of stage I-II NSCLC, with a two-tier screening method.

## RESULTS

### Included studies

**Figure 1:**
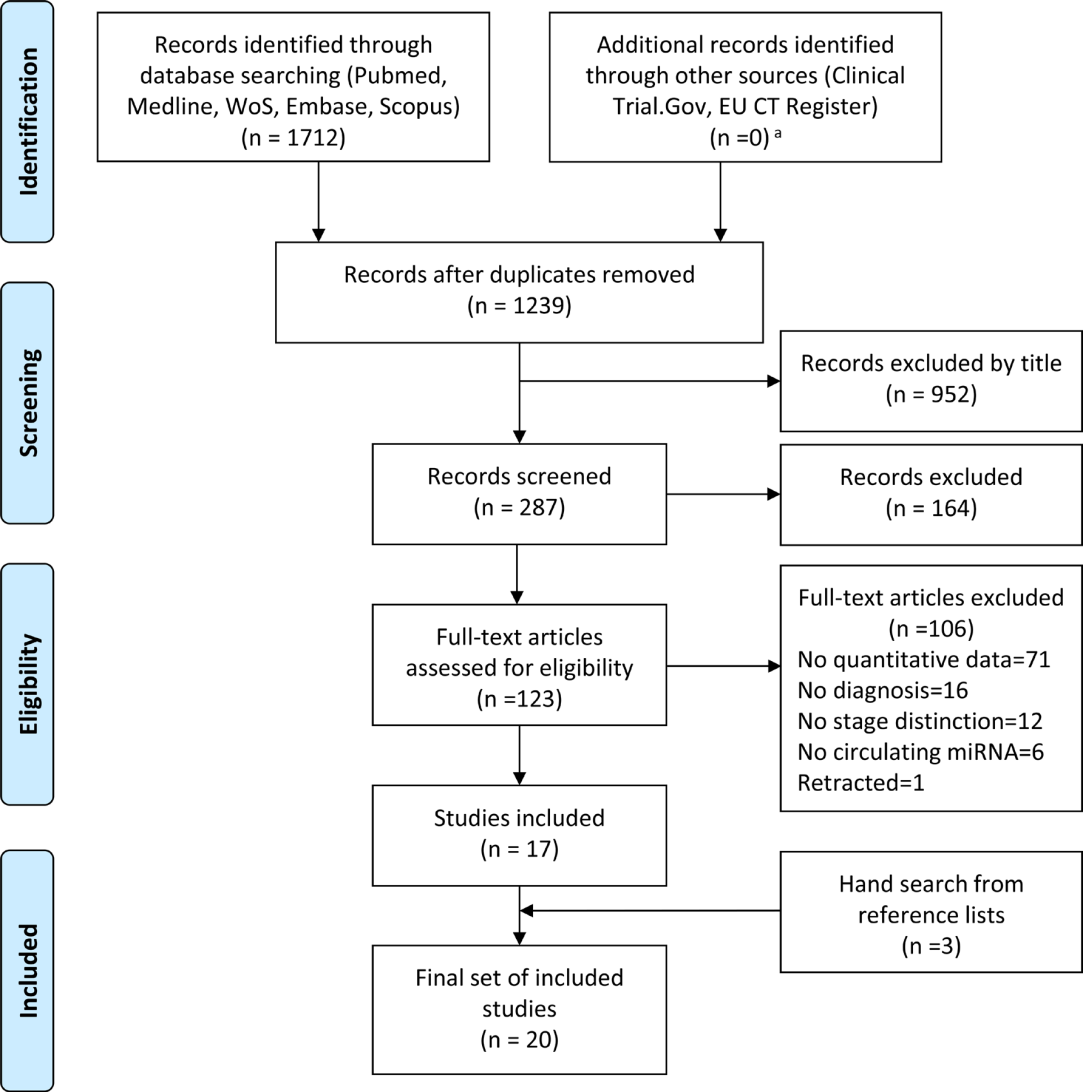
PRISMA flow diagram illustrating the study selection process From the 1712 initially identified studies, duplicates were removed and records were screened by title, abstract, full text, leading to inclusion of 17 studies. Manual search of these papers led to inclusion of 3 further studies, for a total of 20 studies finally included in our review. ^a^Among completed studies, no protocol satisfying the inclusion criteria was retrieved. Protocols in the recruiting stage were excluded.

Our literature search identified a total of 1712 articles, from which duplicates were removed, yielding 1239 papers. After reviewing titles, abstracts and full texts, 17 papers fulfilling our search criteria were finally included. Manual search of the bibliography of these papers led to include 3 additional records, yielding a total of 20 articles (Figure 1). Among these, 8 papers studied single miRNAs only, 6 explored both single miRNAs and panels, and 6 focused on miRNA panels only. For the 20 studies included in the review, Supplementary Table 1A indicates the main characteristics of patients and controls, and the investigated individual miRNAs or panels; Supplementary Table 1B provides information on methods used for miRNA quantification.

The selected studies, all published in the years 2011–2017, included 2119 individuals in total (1110 NSCLC patients and 1009 controls). The sample size ranged between 11 and 126 for NSCLC cohorts and between 11 and 110 for controls. The median sample size was 56 patients (interquartile range 30–79) with 1.1 case/control ratio. In all selected papers the sample mean age ranged 60–65 years, except in the study by Shi and colleagues that was carried out in a younger patient group (patient mean age, 50) [41].

Of the 20 studies (10 from China, 4 from USA, 2 from Italy and 1 each from Poland, Norway, Russia and France), 8 were conducted on Caucasian patients (2 studies included African American subjects), 7 on Asian patients and 5 did not provide information on ethnicity (Supplementary Table 1A).

The NSCLC patient groups differed by clinicopathological status across the studies and some relevant data were missing. Regarding the patients’ smoking status and comorbidities, four studies did not report any data on smoking [41–44]; two studies included only smokers (with > 20 mean pack-years) [26, 30]; five studies included ≥ 85% of smokers among NSCLC patients [27, 29, 45–47]. Fourteen of the 20 studies provided no information on comorbidity of the patient cohort; the other 6 studies indicated that patients had no history of other cancers (Supplementary Table 1A) [26, 48–52]. A mix of the two main subtypes of NSCLC, AC and SCC, was present in all the selected papers, however only in 11 studies the accuracy of the miRNA profile of NSCLC was separately evaluated for AC and SCC [26, 29, 30, 41, 44, 46, 48–50, 53, 54]. The composition of control groups was also varied (Supplementary Table 1A). Four studies [30, 44, 53, 55] provided no medical information on the control group. In 4 studies, history of no tumor and negative chest imaging (X-rays or CT scan) were used to identify healthy controls [26, 46, 47, 51]. In the other 12 studies, individuals broadly defined “healthy subjects” or “non-neoplastic subjects” based on medical history, served as controls; 3 of these studies included patients with chronic obstructive pulmonary disease (COPD) [29, 45, 47] and 4 studies included controls with benign pulmonary nodules or non-cancerous lung disease [45, 48, 50, 52].

### miRNA extraction

miRNAs were extracted from serum samples in 10 studies, from plasma samples in 9 studies, from whole blood in 1 study (Supplementary Table 1B). A training and a validation set were both described in 8 studies; of these, 4 reported two different procedures to quantify miRNAs in the training and validation sets. For miRNA extraction (Supplementary Table 1B), the mirVana PARIS RNA kit (Ambion, ThermoFisher) was used in 7 studies, the miRNeasy mini kit (Qiagen) in 3 studies, and in one study each the miRCURY RNA isolation kit (Exiqon), the RNA extraction kit (Applied Biosystems, AB), the NucleoSpin miRNA Plasma kit (Macherey-Nagel) were used. In the study by Yuxia et al. [42], RNA extraction was not performed, whereas 2 studies used phenol and guanidine isothiocyanate reagents only [27, 49]. Addition of spike-ins as a quality control step was reported in 5 papers (Supplementary Table 1B).

### miRNA retrotranscription and quantification

In 13 studies “Taqman” stem&loop primers and kits (AB) were used for retrotranscription, combined with two quantification methods [Taqman Low Density Arrays microRNA signature panel (TLDA, AB)] or another array -6 papers- and/or probe based relative quantitative PCR (qPCR) -12 papers-, as detailed in Supplementary Table 1B. Absolute miRNA quantification by Droplet Digital PCR (ddPCR) was performed in only 1 study, after retrotranscription with stem&loop primers [28]. In 3 studies, qPCR based on intercalating dyes was used for miRNA quantification. In 3 studies insufficient details of the procedures were provided [29, 41, 48]; (Supplementary Table 1B).

### Normalization

As shown in Supplementary Table 1B, in the 6 studies using TLDA for quantification, the data were normalized with a geometric mean of different miRNAs, or with global normalization, or with quantile normalization. In the 14 studies using qPCR quantification, a single endogenous reference molecule (miR-16 or U6) was used in 7 studies; 4 studies used means of at least two endogenous reference genes; 2 studies used a single exogenous spike-in; 1 study did not provide details about normalization [42] (Supplementary Table 1B).

### Individual miRNAs

In the 20 selected studies, altogether 27 miRNAs were individually reported (Table 1). Overall the studies were of medium quality as assessed by Quality Assessment of Diagnostic Accuracy Studies (QUADAS-2) checklist, where “patient selection” and “index test” resulted the most critical domains. The diagnostic performance of individual miRNAs varied widely: sensitivity ranged between 30.4% and 96.1%, specificity between 38.2% and 100%; moreover, for some miRNAs (miR-223, miR-21, miR-145, miR-125b) the AUC differed substantially between independent studies (Table 1), suggesting that variability of patients, controls or methods may affect miRNA levels.

**Table 1:** Sensitivity, specificity and AUC of the 27 individual miRNAs described in the selected studies

miRNA	Reference	N	Sensitivity (%)	Specificity (%)	AUC
**223**	Geng et al., 2014 [48]	186	**87.0**	86.0	**0.94**
	Zhang et al, 2017 [51]	172	69.8	84.3	0.81
486*	Li et al., 2015 [43]	22	91.0	82.0	0.93
**20a**	Geng et al., 2014 [48]	186	**83.0**	81.0	**0.89**
	Zhang et al, 2017 [51]	172	79.8	88.0	089
**448**	Powrozek et al., 2016 [46]	114	**85.0**	77.0	**0.89**
21*	Sun et al., 2016 [55]	82	--	--	0.88
	Zhang et al, 2017 [51]	172	77.5	85.5	0.84
	Geng et al., 2014 [48]	186	67.0	68.0	0.77
21-5p*	Ma et al., 2013 [28]	74	--	--	0.79
**145**	Zhang et al, 2017 [51]	172	**80.6**	89.2	**0.89**
	Geng et al., 2014 [48]	186	70.0	68.0	0.77
141	Nadal et al., 2015 [45]	135	--	--	0.88
193b	Nadal et al., 2015 [45]	135	--	--	0.86
200b	Nadal et al., 2015 [45]	135	--	--	0.85
126*	Zhu et al., 2016 [52]	127	62.1	97.5	0.85
301	Nadal et al., 2015 [45]	135	--	--	0.84
328	Ulivi et al., 2013 [49]	78	--	--	0.82
4478	Powrozek et al., 2016 [46]	114	75.0	68.4	0.82
125b	Shi et al., 2017 [41]	210	30.4	83.9^$^	0.81^$^
	Yuxia et al., 2012 [42]	186	96.1	38.2	0.66
**1244**	Wang W et al., 2016 [53]	69	53.8	**100.0**	0.80
182	Zhu et al., 2016 [52]	127	67.8	85.0	0.78
425-3p	Wang Y et al., 2016 [50]	173	67.1	68.1	0.73
**628-3p**	Wang Y et al., 2016 [50]	173	42.7	**91.2**	0.73
**29c**	Zhu et al., 2014 [44]	84	50.0	**95.8**	0.73
429	Zhu et al., 2014 [44]	84	94.4	41.7	0.72
22	Shi et al., 2017 [41]	210	43.5	86.3^$^	0.72^$^
335-3p	Ma et al., 2013 [28]	74	--	--	0.71
532	Wang Y et al., 2016 [50]	173	53.7	80.2	0.66
**210**	Zhu et al., 2016 [52]	127	35.6	**100.0**	0.65
183	Zhu et al., 2016 [52]	127	41.4	82.5	0.64
15b*	Shi et al., 2017 [41]	210	41.3	82.4^$^	0.62^$^

N: sample size.

*asterisk denotes miRNA influenced by hemolysis; miRNA was considered hemolysis-influenced when documented in three or more relevant independent studies reporting hemolysis-induced miRNA dysregulation (see Supplementary Table 2).

miRNAs shown in **bold** are those uninfluenced by hemolysis (see Additional Table 2) and with high sensitivity (> 80%) and high AUC (> 0.80) reported in at least one study shown in Reference column.

miRNAs shown in **bold underlined** are those uninfluenced by hemolysis (see Additional Table 2) and with high specificity (> 90%) reported in at least one study shown in Reference column.

^$^Data are calculated among the total sample, that includes advanced stages (III and IV).

### Identification of highly sensitive and highly specific miRNAs

Aiming to identify within Table 1 the individual miRNAs suitable for possible clinical application as non-invasive screening tool, we needed first to eliminate miRNAs influenced by hemolysis, a major source of bias. Therefore, we decided to exclude those miRNAs described as influenced by hemolysis in 3 or more of the relevant independent studies reporting hemolysis-induced miRNA dysregulation [56–63]. Accordingly, five miRNAs listed in Table 1 were excluded from possible clinical use: miR-486, miR-21, miR-21-5p, miR-126, miR-15b (see Supplementary Table 2). Among all miRNAs listed in Table 1 and considered unaffected by hemolysis, only miR-223, miR-20a, miR-448 and miR-145 displayed AUC value > 0.80 and sensitivity > 80% as stage I-II NSCLC biomarkers in at least one study. Moreover, miR-628-3p, miR-29c, miR-210 and miR-1244, despite low sensitivity and modest AUC value, showed specificity > 90% in at least one study. For these two sets of miRNAs, highly sensitive and highly specific respectively, the diagnostic accuracy data are summarized in Table 2. The seven studies analyzing these miRNAs [44, 46, 48, 50–53] are of medium overall quality according to the QUADAS-2 checklist; they included 649 NSCLC patients (median, 87 patients per study), predominantly smokers (62%) and represent 58% of the 1110 patients evaluated overall in the 20 selected studies. The miRNAs shown in Table 2 have important biological functions related to tumorigenesis; although the analysis of these functions is beyond the purpose of this review, they are briefly described in Supplementary File 1.

Table 2miRNAs with high sensitivity and high AUC (a), and miRNAs with high specificity (b).

**Table:** a) miRNAs with sensitivity > 80% and AUC > 0.80

miRNA	Sensitivity (%)	AUC	Specificity (%)	Reference
miR-223	87.0	0.94	86.0	Geng et al., 2014 [48]
miR-20a	83.0	0.89	81.0	Geng et al., 2014 [48]
miR-448	85.0	0.89	77.0	Powrozek et al., 2016 [46]
miR-145	80.6	0.89	89.2	Zhang et al., 2017 [51]

**Table:** b) miRNAs with specificity > 90%

miRNA	Sensitivity (%)	AUC	Specificity (%)	Reference
miR-628-3p	42.7	0.73	91.2	Wang Y et al., 2016 [50]
miR-29c	50.0	0.73	95.8	Zhu et al., 2014 [44]
miR-210	35.6	0.65	100.0	Zhu et al., 2016 [52]
miR-1244	53.8	0.80	100.0	Wang W et al., 2016 [53]

### miRNA panels

In the 20 selected studies, 12 miRNA panels featuring high sensitivity (> 80%) and/or high AUC (> 0.80) as stage I-II NSCLC biomarkers were reported (Table 3). Five of these panels showed AUC> 0.90 and seven had AUC between 0.80 and 0.90; however some panels included miRNAs documented to be influenced by hemolysis (Table 3).

**Table 3:** Sensitivity (Se), specificity (Sp) and AUC of miRNA panels described in the selected studies

	miRNA Panel	Reference	*N*	Se(%)	Sp(%)	AUC
1	miR-141, miR-200b, miR-193b, miR-301	Nadal et al., 2015 [45]	135	N.R.	N.R.	0,99
2^1^	24 miRNAs*	Wozniak et al., 2015 [54]	121	N.R.	N.R.	0,98
2 bis^2^	24 miRNAs*	Wozniak et al., 2015 [54]	149	N.R.	N.R.	0,96
3	miR-182*,* miR-183*,* **miR-210***, miR-126*, CEA	Zhu et al., 2016 [52]	127	88,5	92,5	0,98
4	miR-532*,* **miR-628-3p***,* miR-425-3p	Wang Y. et al. 2016 [50]	173	91,5	97,8	0,97
**5**	**miR-448**, miR-4478	Powrozek et al., 2016 [46]	114	90	76.3	0,90
**6**	**miR-145**, **miR-20a***, miR-21,* miR-223	Zhang et al., 2017 [51]	172	81,8	90,1	0,90
7	34 miRNAs**	Bianchi et al., 2011 [26]	52	59	90	0,89
8	miR-125b, miR-200b, miR-34b, miR-203, miR-205, miR-429	Halvorsen et al., 2016 [47]	158	85	74	0,88
9	*miR-21-5p,* miR-335-3p	Ma et al., 2013 [28]	74	N.R.	N.R.	0,86
10^1^	12 miRNAs***	Sanfiorenzo et al., 2013 [29]	33	N.R.	N.R.	0,85
10 bis^2^	12 miRNAs***	Sanfiorenzo et al., 2013 [29]	42	N.R.	N.R.	0,81
11	miR-1254, miR-574-5p	Foss et al., 2011 [27]	53	73	71	0,75
12^2^	*mir-21, miR-126,* **miR-210***, miR-486-5p*	Shen et al., 2011 [30]	44	73,3	96,5	N.R.
12 bis^1^	*mir-21, miR-126,* **miR-210***, miR-486-5p*	Shen et al., 2011 [30]	44	86,7	96,5	N.R.

N: sample size.

N.R.: Not Reported.

Panels are listed in decreasing order of AUC value. The studies by Wozniak et al., Sanfiorenzo et al., Shen et al. [54, 29, 30], separately described findings in stage I^2^ and stage II^1^ non-small cell lung cancer.

*let-7c**,** miR-122, miR-182, miR193a-5p, miR-200c, miR-203, miR-218, miR-155, let-7b, miR-411, miR-450b-5p, miR-485-3p, miR-519a, miR-642, miR-517b, miR-520f, miR-206, miR-566, miR-661, miR-340, miR-1243, miR-720, miR-543, miR-1267.

**miR-92a, miR-484*, miR-486-5p,* miR-328, miR-191, miR-376a, miR-342-3p, miR-331-3p*,* miR-30c, miR-28-5p, miR-98*, miR-17,* miR-26b*,* miR-374a*,* miR-30b*,* miR-26a*,* miR-142-3p*, miR-103, miR-126,* let-7a*,* let-7d*,* let-7b*,* miR-32*,* miR-133b, miR-566, mir-432, miR-223*, miR-29a,* miR-148a, miR-142-5p*,* miR-22*,* miR-148b*,* miR-140-5p**,** miR-139-5p.

***miR-155-5p, **miR-20a-5p**, miR-25-3p*,* miR-296-5p*,* miR-191-5p*, miR-126-3p,* miR-223-3p*,* miR-152-3p, **miR-145-5p**, miR-199a-5p*, miR-24-3p***,** and let-7f-5p.

miRNAs influenced by hemolysis are indicated in *italics*; miRNA was considered hemolysis-influenced if influence was documented in three or more relevant independent studies reporting hemolysis-induced miRNA dysregulation (see Supplementary Table 2).

miRNAs indicated in **bold** are also accurate individual predictors, included in Table 2.

Table 4 illustrates miRNAs that were described (either individually or within miRNA panels) as biomarkers of stage I-II NSCLC in more than one of the selected studies.

**Table 4:** miRNAs indicated as stage I-II NSCLC biomarkers in more than one of the selected studies

miRNA	Number of studies^a^	References
miR-21	4	Sun et al., 2016 [55]; Zhang et al., 2017 [51]; Geng et al., 2014 [48]; Shen et al., 2011 [30]*
**miR-223**	4	Zhang et al., 2017 [51]; Geng et al., 2014 [48]; Sanfiorenzo et al., 2013 [29]*; Bianchi et al., 2011 [26]*
miR-126	4	Zhu et al., 2016 [52]; Sanfiorenzo et al., 2013 [29]*; Shen et al., 2011 [30]*; Bianchi et al., 2011 [26]*
**miR-20a**	3	Zhang et al., 2017 [51]; Geng et al., 2014 [48]; Sanfiorenzo et al., 2013 [29]*
**miR-145**	3	Zhang et al., 2017 [51]; Geng et al., 2014 [48]; Sanfiorenzo et al., 2013 [29]*
miR-125b	3	Shi et al., 2017 [41]; Halvorsen et al., 2016 [47]*; Yuxia et al., 2012 [52]
miR-486	3	Li et al., 2015 [77]; Shen et al., 2011 [30]*; Bianchi et al., 2011 [26]*
miR-155	3	Geng et al., 2014 [48]; Wozniak et al., 2015 [54]*; Sanfiorenzo et al., 2013 [29]*
miR-200b	2	Halvorsen et al., 2016 [47]*; Nadal et al., 2015 [45]*
miR-328	2	Ulivi et al., 2013 [49]; Bianchi et al., 2011 [26]*
miR-182	2	Zhu et al., 2016 [52]; Wozniak et al., 2015 [54]*
miR-429	2	Halvorsen et al., 2016 [47]*; Zhu et al., 2014 [44]
**miR-210**	2	Zhu et al., 2016 [52]; Shen et al., 2011 [30]*
miR-22	2	Shi et al., 2017 [41]; Bianchi et al., 2011 [26]*
miR-203	2	Halvorsen et al., 2016 [47]*; Wozniak et al., 2015 [54]*
let-7b	2	Wozniak et al., 2015 [54]*; Bianchi et al., 2011 [26]*
miR-566	2	Wozniak et al., 2015 [54]*; Bianchi et al., 2011 [26]*
miR-191	2	Sanfiorenzo et al., 2013 [29]*; Bianchi et al., 2011 [26]*

^a^number of reviewed studies indicating the specified miRNA as stage I-II NSCLC biomarker.

*Asterisk denotes panel including the specified miRNA.

miRNAs indicated in **bold** are included in our two-step screening (Table 2).

### miRNAs and NSCLC subtypes

None of the 20 studies included in this review separately evaluated miRNA signatures in SCC or AC. However, 11 of the 20 selected studies evaluated the performance of circulating miRNAs in distinguishing NSCLC subtypes, and the investigated individual miRNAs and miRNA panels completely differed across the studies. As summarized in Table 5, the proposed miRNA signatures revealed: greater accuracy in identifying SCC than AC in 7 studies [26, 29, 41, 46, 48, 49, 53]; similar accuracy in 2 studies [44, 54]; higher sensitivity for diagnosing AC in one study [30]. Wang et al. [50] reported that circulating levels of miR-425-3p and miR-628-3p were significantly higher in AC than SCC, while miR-532 was significantly lower in AC than SCC (Table 5). The AUCs for the miRNAs proposed as subtype-specific biomarkers were reported only in 4 studies [26, 48, 53, 54]. Altogether these findings show no consistent alterations of circulating miRNAs that may more accurately identify AC or SCC.

**Table 5:** Characteristics of the 11 included studies evaluating the performance of circulating miRNAs in distinguishing NSCLC subtypes

Reference	Year	Sample ethnicity	Sample Sizea		NSCLCd Stage	miRNAs examined	AUC^e^ in discriminating NSCLC subtype from controls for the examined miRNAs		Comments on miRNA performance
			Pt^b^	C^c^			AC^f^ (*n*)	SCC^g^ (*n*)	
**Bianchi et al.** **[26]**	**2011**	Caucasian	22^h^	30	I	Panel of 34 miRNAs*	(*n =* 22) 0.85	(*n =* 12) 0.94^h^	The panel distinguished better SCCs than ACs from controls; however, the SCCs were stage II-IV cases. Sample size was small.
**Geng et al.** **[48]**	**2014**	Asian	126	60	I-II	5 miRNAs: miR-20a miR-223 miR-21 miR-155 miR-145	(*n =* 45) 0.90 0.91 0.63 0.93 0.77	(*n* = 64) 0.98 0.98 0.97 0.96 0.97	All 5 miRNAs differentiated NSCLC from controls with greater accuracy in SCCs. 17 cases had histology other than AC or SCC.
**Powrozek et al.** **[46]**	**2016**	Caucasian	29^i^	85	I-II	2 individual miRNAs: miR-448, miR-4478; combination of both miRNAs	(*n =* 30) NA^l^	(*n =* 35) NA	Both miRNAs overexpressed in NSCLC plasma samples relative to control. miR-4478 expression was higher in SCC than in AC patients (*p* < 0.043). Analysis of miRNA performance included 36 cases with stage >II.
**Sanfiorenzo et al.** **[29]**	**2013**	NA	35^i^	20	I-II	Panel of 12 miRNAs**	(*n =* 27) NA	(*n =* 25) NA	Panel distinguished NSCLC patients from controls (AUC=0.81). In SCC compared to AC, higher plasma levels of miR-20a-5p (*p* = 0.034) and miR-25-3p (*p* = 0.013), along with lower levels of miR-191-5p (*p* = 0.008) were found. Analysis of miRNA performance included 17 cases with stage >II.
**Shen et al.** **[30]**	**2011**	African American, Caucasian	30^i^	29	I-II	miR-21, miR-126, miR-210, miR-486-5p	(*n =* 24) NA	(*n =* 34) NA	Diagnostic sensitivity of the composite panel in distinguishing stage I NSCLC from controls was 73.3%. Analysis of miRNA performance in diagnosing subtypes included 28 cases with stage >II and showed higher sensitivity for diagnosing ACs (91.7%) than SCCs (82.3%) (*p* < 0.05).
**Shi et al.** **[41]**	**2017**	NA	46^i^	45	I-II	miR-22, miR-125b, miR-15b	(*n =* 69) NA	(*n =* 51) NA	Serum levels of the three miRNAs significantly altered in NSCLC cases compared to controls. Diagnostic sensitivity of miR-125b was significantly higher for ACs than SCCs (*p* = 0.021). Analysis of miRNA performance included 74 cases with stage >II.
**Ulivi et al.** **[49]**	**2013**	Caucasian	54^i^	24	I-II	miR-328	(*n =* 63) NA	(*n =* 22) NA	miR-328 discriminated well between stage I-II NSCLC and controls (AUC = 0.82). Analysis of miRNA performance for subtypes, conducted in 86 NSCLCs (63 ACs; 22 SCC; 1 sarcomatoid), 32 of which were in stage > II, indicated significantly higher expression of miR-423 in SCCs than in ACs. The miRNA analyses were performed in whole blood specimens.
**Wang Y. et al.** **[50]**	**2016**	Asian	82	91	I-II	miR-532, miR-628, miR-425-3p	(*n =* 40) NA	(*n =* 39) NA	Combination of the three miRNAs discriminated well NSCLC from control plasma samples (AUC = 0.97). Evaluation of miRNA performance was conducted in 40 ACs and 39 SCCs. Plasma levels of miR-425-3p (*p* = 0.04) and of miR-628-3p (*p* = 0.015) were significantly higher in AC than SCC. miR-532 was significantly lower in AC than SCC (*p* < 0.001).
**Wang W. et al.** **[53]**	**2016**	NA	54^i^	15	I-II	miR-1244	(*n =* 26) 0.79	(*n =* 17) 0.85	For miR-1244 the AUC was higher in SCC than AC. AUC was assessed on serum samples of 43 NSCLCs (26 ACs; 17 SCCs), 17 of which were in stage > II.
**Wozniak et al.** **[54]**	**2015**	Caucasian	70^i^	100	I-II	Panel of 24 miRNAs***	(n, NA) 0.94	(n, NA) 0.96	Panel showed similar accuracy for distinguishing AC and SCC from controls. AUC for the panel was assessed in 70 NSCLCs [a sub-cohort of 100 NSCLCs (35 ACs; 65 SCCs), 30 of which were in stage >II].
**Zhu et al.** **[44]**	**2014**	Asian	36^i^	48	I	miR-29c, miR-93, miR-429	(*n* =34) NA	(*n =* 36) NA	The evaluation of 70 NSCLCs (34 ACs; 36 SCC), 34 of which were in stage II–IV, showed non-significant difference of serum miR-29c (*p* = 0.232) and miR-429 (*p* = 0.811) between AC and SCC.

^a^The sample refers to stage I and II NSCLC.

^b^Pt=Patients; ^c^C=Controls.

^d^NSCLC: non-small cell lung cancer.

^e^AUC=area under the curve.

^f^AC: adenocarcinoma.

^g^SCC=squamous cell carcinoma.

^h^SCCs were 12 additional cases with stage II-IV disease.

^i^Analysis of miRNA performance included also cases with stage > II^.^

^l^NA: Not available.

*miR-92a, miR-484, miR-486-5p, miR-328, miR-191, miR-376a, miR-342-3p, miR-331-3p, miR-30c, miR-28-5p, miR-98, miR-17, miR-26b, miR-374a, miR-30b, miR-26a, miR-142-3p, miR-103, miR-126, let-7a, let-7d, let-7b, miR-32, miR-133b, miR-566.

**miR-155-5p, miR-20a-5p, miR-25-3p, miR-296-5p, miR-191-5p, miR-126-3p, miR-223-3p, miR-152-3p, miR-145-5p, miR-199a-5p, miR-24-3p, and let-7f-5p.

***let-7c, miR-122, miR-182, miR-193a-5p, miR-200c, miR-203, miR218, miR-155, let-7b, miR-411, miR-450b-5p, miR-485-3p, miR-519a, miR-642, miR-517b, miR-520f, miR-206, miR-566, miR-661, miR-340, miR-1241, miR-720, miR-543, miR-1267.

### Proposal of a two-step screening with miRNAs

Based on the critique of the reviewed studies, we propose a model for screening of stage I-II NSCLC, using the two above indicated sets of individual miRNAs with the highest sensitivity/specificity (Table 2), that were selected as detailed in the Methods section. Panels of miRNAs were arbitrarily excluded from this model as the AUC or specificity data were based on the panels, and to simplify its possible clinical application. Accordingly, screening with miRNAs should be carried out in two steps. The four miRNAs with high sensitivity (miR-223, miR-20a, miR-448 and miR-145) should be used for the first screening step (Test 1), and the four miRNAs with high specificity (miR-628-3p, miR-29c, miR-210 and miR-1224) for the second step (Test 2). In this model the two panels of miRNAs are combined in series, and Test 2 is run only if Test 1 is positive, as described in Supplementary File 2.

The final estimated performance of these miRNAs for the two-step screening of serum samples is overall sensitivity of 91.6% and overall specificity of 93.4%. The selected two sets of miRNAs with highest sensitivity/specificity are intended for preliminary screening of the general population at high risk of lung cancer, dominated by smokers. Subjects positive to miRNA screening should be offered low-dose CT-screening, thus possibly reducing the logistic/economic burden and harms of upfront CT-screening [12,14].

## DISCUSSION

Diagnosing lung cancer at an early stage is a major clinical concern that in recent years has stimulated extensive research on non-invasive screening methods, including miRNAs as lung cancer biomarkers in circulating body fluids. Dysregulated miRNA profiles in cell-free blood were shown to indicate the presence of lung cancer many months ahead of the occurrence of symptoms [64], and even before the disease was detected by CT screening [26, 65]. Therefore, miRNAs are potentially interesting biomarkers for screening of lung cancer [66]. According to “Medline Trend”, the number of publications on the topic “miRNA and NSCLC” has dramatically increased in the last 10 years, however the plethora of circulating miRNA profiles proposed as lung cancer signatures are inconsistent. Six systematic reviews [34–37, 39, 40] have summarized the main findings of these studies, but have failed to clearly identify circulating miRNAs possessing high proficiency specifically for the diagnosis of stage I-II NSCLCs, which are the cancers potentially amenable to radical cure. Considering that miRNA signatures of early and late lung cancer stages frequently differ [50, 64, 67–69], we exclusively reviewed papers reporting miRNAs biomarkers of stage I-II NSCLC. We focused on miRNA molecules of high diagnostic accuracy and whose measurement is scarcely influenced by hemolysis. Among the initially retrieved 1712 papers fulfilling the search criteria, we only found 20 studies clearly reporting quantitative data on miRNA diagnostic proficiency specifically for stage I-II NSCLC. Our review confirms the variability of miRNAs proposed by many authors as lung cancer signatures. Notably, there were only 18 miRNAs identified as biomarkers of stage I-II NSCLC in more than one published paper (Table 4).

For the 20 selected studies we highlighted demographics, clinicopathological characteristics and smoking habit of patients and controls; moreover, we evaluated the main pre-analytical and analytical variables known to influence circulating miRNA levels. Notably, the training set in the selected studies consisted of a median of only 56 NSCLC patients, meaning that the training sample frequently was of smaller size than that suggested by guidelines for studies of biomarkers for early detection of cancer [70]. Moreover, in some of the selected studies the training sample appears definitely undersized if one considers the rather low precision of miRNA assays [63] and the expected diversity of miRNA signatures due to molecular heterogeneity of NSCLC subtypes [36, 71–74]. Population ethnicity has been suggested as a potential source of miRNA level variability and of inconsistent miRNA signatures of lung cancer [77]. However, Shen et al. found no association between changes in circulating miRNA levels and patient ethnic group (African-American or Caucasian) [43]. As regards gender and age, several papers have documented that these variables do not significantly impact on lung cancer miRNA signatures [30, 44, 50, 75, 76].

The proportion of smokers varied among the studies and sometimes markedly differed between cases and controls within the same study. In 5 of the 20 papers, cigarette smoking data were not reported, an important lack of information because some circulating miRNAs are significantly dysregulated by smoking [34, 77, 78].

Across the 20 studies, the control groups were also very different. The majority of studies defined the control group as “healthy subjects” not otherwise specified, or “non-neoplastic subjects” based on medical history. Notably, the composition of control groups is a critical issue, because diseases of liver, heart, prostate and various other comorbidities in the control group may influence the diagnostic sensitivity and specificity of miRNA candidate biomarkers [79–81]. Only 4 of 20 studies subdivided control patients by comorbidity: benign lung nodules, COPD, noncancerous disease, smoker [29, 48, 50, 52]. It is debated if “controls” for lung cancer patients should be age-matched “healthy subjects” or subjects with a history of smoking, and whether COPD patients should be included as controls. Because levels of miRNA relevant for lung cancer may be altered in smokers [34, 77] and in COPD patients [82–84], the control group composition in terms of smoking pack/years and COPD prevalence may bias the accuracy of miRNAs selected as lung cancer biomarkers. In order to avoid a COPD-based miRNA signature, in the studies by Sanfiorenzo et al. [29] and Halvorsen et al. [47], non-neoplastic COPD patients were used as controls.

Elegant experimental studies have shown that miRNAs derived from cancer tissue can enter the circulation [24]. Moreover, in lung cancer patients several overexpressed circulating miRNAs (miR-21, miR-24, miR-145, miR-20a, miR-223, miR-486, miR-574-5p, miR-1825, miR-205, miR-19a, miR-19-b, miR-30b) were generally reduced a few days after tumor resection, strongly suggesting that these molecules are of tumor origin or tumor-induced [43, 51, 75, 85, 86]. It is therefore reasonable to assume that at least some of the aberrantly expressed miRNAs in the blood of lung cancer patients are genuine biomarkers of the tumor. The measurement of circulating miRNAs faces numerous technical challenges and may be biased by multiple factors, partly explaining the inconsistency of published miRNA profiles of lung cancer [51]. Because several circulating miRNAs are blood-cell derived [56, 59–62, 87], spurious miRNA level dysregulations that may result from platelet contamination and red blood cell lysis in plasma/serum samples are a major concern. Our review suggests that potential bias of hemolysis on miRNA levels has often been underestimated, as only in 2 of the 20 reviewed studies was hemolysis of specimens ruled out [29, 54]. In order to avoid spurious effects of undetected hemolysis of samples, in agreement with Pritchard and collaborators [88], we suggest that miRNAs influenced by hemolysis should preferably not be used as NSCLC biomarkers.

For analysis of circulating miRNAs, both serum and plasma are acceptable sample types, and a good correlation between serum and plasma miRNA determinations has been documented [89]. However, serum and plasma determinations cannot be automatically interchanged, because differences in specimen preparation and/or measurement platform are known to influence the results. As an example, in normal subjects miR-15b and miR-16 showed higher concentrations in plasma relative to serum in one study [63], while the concentration of the same two miRNAs was higher in serum relative to plasma in another independent study using a different platform [90].

It is currently debated whether serum or plasma should be used for circulating miRNAs determination; mirroring this uncertainty, 10 of the 20 selected studies were performed with serum samples and 9 with plasma. Serum has not been generically recommended over plasma as a sample type [87]; however, serum has less platelet contamination than plasma, and this may decrease bias in miRNA determination [63]. Regarding the method for miRNA quantification, all the reviewed studies except that of Ma et al. [28] used qPCR platform and performed normalization of results predominantly with endogenous miR-16 and U6, or with spike-ins. The normalization step is likely to contribute to the scarce reproducibility of miRNA determinations, as reported by others [33]. Normalization with miR-16 can be criticized because this miRNA has been described as a lung cancer biomarker itself [91–93]. U6, a small nuclear RNA, was shown to fluctuate markedly across samples [94], and such variability may contribute to inconsistency of miRNA findings. For miRNA measurement Ma et al. [28] used ddPCR, a recently introduced technique reported to be advantageous over qPCR (greater precision; no need to normalize results; higher sensitivity to low-level miRNA expression) [28, 95]. Altogether, these considerations underscore the importance of knowing the miRNA quantification procedure details, to allow reproducibility of methods and external validation of studies.

Distinguishing between the AC and SCC lung cancer subtypes on the basis of specific circulating miRNAs’ aberrant expression may provide important information, relevant both for understanding the subtypes’ pathogenesis and for tailored selection of cytotoxic chemotherapy in NSCLC without a driver mutation [96]. Moreover, although histology and immunohistochemistry (IHC) currently are the gold standards for NSCLC diagnosis, the subtype classification of difficult cases (scarce biopsy sample; hazardous/difficult biopsy; uncertain IHC) could be facilitated if subtype-specific circulating miRNA signatures were available. Few studies have been conducted in stage I-II NSCLC patients to identify circulating miRNA profiles than may be more accurate for either AC or SCC. In our systematic review we only found 11 studies that separately analyzed AC and SCC cases [26, 29, 30, 41, 44, 46, 48–50, 53, 54], and none of these provided convincing evidence that a specific miRNA signature exists for each of the two subtypes. A notable methodological weakness in 9 of these 11 studies [26, 29, 30, 41, 44, 46, 48–50, 53, 54] is the inclusion of many lung cancers in advanced stage (stage > II) in the subtype analysis, likely to compensate for small sample size of the AC and SCC sub-cohorts. Of note, Bianchi et al. [26], Geng et al. [48], Powrozek et al. [46], Sanfiorenzo et al. [29], Shi et al. [41], Ulivi et al. [49] and Wang et al. [53] proposed very different miRNA signatures of early stage NSCLC, yet all these signatures better differentiated SCC than AC from controls. Altogether, the available data are insufficient to define serum/plasma miRNA profiles that may reliably discriminate between AC and SCC in stage I-II lung cancer.

### Strengths and limitations

A strength of this review is the critique focused on circulating miRNA biomarkers of stage I-II NSCLC, the disease stages often amenable to radical cure and for which non-invasive screening by miRNAs may be proposed. Another strength is the assessment of factors potentially influencing miRNA levels and the evaluation of miRNAs’ accuracy as stage I-II NSCLC biomarkers by quantitative data (sensitivity, specificity, AUC).

This review has important limitations. First, miRNA signatures of NSCLC may be biased by pre-analytical and analytical factors, and by clinicopathological features of patients and controls. Second, in many of the selected papers the validation sample was relatively small (median, 56 patients), with limited power to correctly identify the miRNA signature of stage I-II NSCLC. Third, lack of methodological details in some studies prevented thorough evaluation of the quality of methodology used. Data on comorbidities, some of which may affect miRNA expression [79–81], were not provided in some papers. Accordingly, at QUADAS-2, “patient selection” and “index test” resulted the most critical domains, and overall the studies were only of medium quality. Another limitation is that the miRNA panels for our two-step model of screening were obtained from studies where only the majority of lung cancer patients (62%) were smokers, while screening for lung cancer is currently recommended exclusively in smokers (11). In this review, we aimed to identify circulating individual miRNAs with sensitivity > 80% and AUC > 0.80 as biomarkers of stage I-II NSCLC, for possible clinical application as non-invasive screening tool. Based on the reviewed studies, we found four individual miRNAs that fulfilled these criteria: miR-223, miR-20a, miR-448 and miR-145; four other miRNAs showed very high specificity (> 90%): miR-628-3p, miR-29c, miR-210 and miR-1244. Among factors potentially affecting circulating miRNAs, the only two that were considered for miRNAs selection were the stage of NSCLC (all studies were stages I-II) and the impact of hemolysis (miRNAs potentially affected by hemolysis were excluded). Other factors, such as smoking habits, age, ethnicity, methodological issues of RNA extraction, could not be controlled because they varied widely among the selected studies.

Screening for lung cancer with circulating miRNAs, preliminary to CT-screening, is a minimally invasive and safe blood test that may offer several advantages over upfront CT-screening: reduction of number of CT-screens (to be performed only in miRNA screening-positive individuals) and of radiation risk; decrease of false-positive CT-screening rate and consequent reduction of complications and costs from futile lung biopsies (12,14). We have proposed a two-step model of miRNA screening for stage I-II NSCLC, based on the measurement of the serum level of the above indicated selected miRNAs (Table 2): the panel of four miRNAs with high sensitivity should be used for the first screening step, and the panel with high specificity for the second step. Based on our model, for the two miRNA panels combined in series for screening of serum samples the estimated performance is overall sensitivity of 91.6% and overall specificity of 93.4%. The estimated diagnostic accuracy of the proposed model is similar to that of the 12 miRNA panels found in the selected papers (Table 3), most of which featured high sensitivity (> 80%) and/or high AUC (> 0.80) as stage I-II NSCLC biomarkers. However, several of these panels contain miRNAs that are not ideal biomarkers; as Table 3 shows, 6 of the 12 panels included miRNAs influenced by hemolysis. Moreover, Nadal et al. and Wozniak et al. provided no sensitivity nor specificity data for their panels [45, 54] and the panels tested by Powrozek et al., Halvorsen et al., and Foss et al. showed modest specificity (76.3%, 74% and 71%, respectively) [27, 46, 47]. The panel proposed by Wang et al. featured high proficiency in diagnosing AC, without containing hemolysis-influenced miRNAs [50].

## MATERIALS AND METHODS

### Search strategy

A systematic review of the scientific literature was conducted using the following key words: [(NSCLC OR Non Small Cell Lung Cancer) AND (lung cancer) AND (miRNA OR MicroRNA) AND (diagnosis)]” on the search engines of the databases “Pubmed”, “Medline”, “Scopus”, “Embase” and “WOS”. The research was first performed on July 21st 2016 and results were regularly updated until April 12th 2017. Including criteria were: i) circulating miRNAs; ii) histologically/cytologically defined NSCLC stage I and/or II (studies of patients with NSCLC at any stage were included only if a sub-analysis for stage I-II was provided); iii) studies reporting quantitative data on the efficacy of specific miRNAs as tools for stage I-II NSCLC screening (sensitivity, specificity and/or AUC); iv) English language. Studies analyzing single miRNAs and/or panels of miRNAs were included. Duplicate publications were eliminated through the Mendeley software [97]. All articles of interest were then evaluated and screened for eligibility by two researchers, independently, and controversies were resolved by consensus. Bibliography of the selected papers was manually examined to retrieve further articles with eligibility criteria.

The protocol was registered at the international prospective register of systematic reviews (PROSPERO, ID: CRD42017056943). The PRISMA statement and the Cochrane Handbook for Diagnostic Test Accuracy Reviews were followed as reference protocol standards.

### Data extraction

From the eligible studies the following information was collected: a) author name, year and country where the study was performed; b) sociodemographic and clinical information on population under study (ethnicity, sample size, age, smoking status, comorbidity, NSCLC stage); c) individual miRNAs and/or miRNA panels under study; d) methodological issues regarding miRNAs extraction [type of specimen (plasma/serum/whole blood), hemolysis assessment, RNA isolation and measurements procedures]; e) quantitative data of diagnostic accuracy (sensitivity, specificity, AUC) for stage I-II NSCLC.

The papers then underwent rigorous critical evaluation, taking into account: i) quality of the study, assessed by the Quality Assessment of Diagnostic Accuracy Studies (QUADAS-2) checklist [98]; ii) factors identified as potentially affecting miRNA quantification (Table 6). Two investigators independently assessed the seven domains of the QUADAS-2. Any discrepancies were resolved through discussion.

**Table 6:** Factors potentially affecting circulating miRNA quantification in NSCLC patients

**Clinicopathological factors**	Ethnicity
	Gender/age
	Smoking status
	Stage of disease (early/advanced)
**Methodological factors**	Type of sample (plasma/serum/whole blood)
	Hemolysis
	RNA extraction method
	Reverse transcription method
	miRNA quantification method
	Normalization

NSCLC: non-small cell lung cancer.

### Selection of circulating miRNAs for a two-step screening preliminary to CT-screening

Within studies with overall satisfactory quality by QUADAS-2, we identified individual miRNAs showing at least in one study high diagnostic proficiency as stage I-II NSCLC biomarkers (arbitrarily stated as sensitivity > 80% and AUC > 0.80, or specificity > 90%) and scarcely influenced by hemolysis according to the pertinent literature [56–63]. Altogether eight individual miRNAs revealed the aforementioned high diagnostic proficiency as stage I-II NSCLC biomarkers (miR-223, miR-20a, miR-448, miR-145, miR-628-3p, miR-29c, miR-210 and miR-1244; Table 2). These miRNAs with the highest sensitivity/specificity can be applied in a mathematical model, that we are here proposing, to estimate their overall sensitivity and specificity for stage I-II NSCLC screening. The model consists of a two-step screening test, first using the panel of selected circulating miRNAs with high sensitivity and high AUC, then the panel of selected miRNAs with high specificity, as illustrated in Supplementary File 2. We arbitrarily excluded miRNA panels from the model since the AUC or specificity data were based on the panels and not individual miRNAs, aiming to simplify possible clinical application of the test. However, for comparison of our model’s diagnostic accuracy, the other miRNA panels included in the review are discussed.

### Statistical analysis

The proposed two-step model for estimating overall sensitivity and specificity of circulating miRNAs to be used for stage I-II NSCLC screening was developed using the formulas described in Supplementary File 2.

## CONCLUSIONS

Several pre-analytical and analytical variables of circulating miRNA measurements, especially hemolysis of samples, may bias the accuracy of miRNAs as biomarkers of stage I-II NSCLC. Evidence-based data are insufficient to reach a robust conclusion as to which circulating miRNAs are the best biomarkers of early lung cancer, and also insufficient to define serum/plasma miRNA profiles that may reliably discriminate between AC and SCC.

Nevertheless, based on critical review of the literature, selected circulating miRNAs that are scarcely influenced by hemolysis could be tested for screening early lung cancer in smokers and former smokers. For our theoretical model of two-step screening for stage I-II NSCLC, first using a panel of miRNAs with high sensitivity and then a panel with high specificity, we estimated overall sensitivity of 91.6% and overall specificity of 93.4%. The circulating miRNAs we selected as potentially valuable biomarkers of early lung cancer based on this review, as well as those described by other authors, require validation in multiple independent studies before they can be proposed for clinical application.

## SUPPLEMENTARY MATERIALS TABLES











## Abbreviations

AB: Applied Biosystems
AC: adenocarcinoma
AUC: area under the curve
COPD: chronic obstructive pulmonary disease
CT: computed tomography
ddPCR: droplet digital PCR
IHC: immunohistochemistry
miRNA: microRNA
NSCLC: Non-small cell lung cancer
qPCR: quantitative (realtime) PCR
QUADAS: Quality Assessment of Diagnostic Accuracy Studies
SCC: squamous cell carcinoma
TLDA: Taqman Low Density Arrays
