# Supplementary material for: Systematic review and critique of circulating miRNAs as biomarkers of stage I-II non-small cell lung cancer
Source: Oncotarget. 2017 Oct 11;8(55):94980–96. doi: 10.18632/oncotarget.21739 (PMC5706930; doi:10.18632/oncotarget.21739)
Supplement: Supplementary file 1 [file oncotarget-08-94980-s001.pdf]

Systematic review and critique of circulating miRNAs as biomarkers of stage I-II non-small cell lung cance

SUPPLEMENTARY MATERIALS

Supplementary File 1: Biological functions of microRNAs proposed as biomarkers for two-step screening of stage I-II NSCLC in the serum. See Supplementary_File_1

Supplementary File 2: Model proposed to estimate the overall sensitivity and specificity of 8 miRNAs used for a two-step screening of stage I-II NSCLC. See Supplementary_File_2

Supplementary Table 1A. Main characteristics of the 20 included studies. See Supplementary_Table_1A

Supplementary Table 1B. Materials and procedures used for miRNA quantification in the 20 included studies. See Supplementary_Table_1B

Supplementary Table 2: Effect of hemolysis on measurement of circulating miRNAs listed in Tables 1 and 3

miRNA	Affected by hemolysis[a] (yes/no)	References[b]
**223**	no	MacLellan et al. [59]
486	yes	MacLellan et al. [59], Landoni et al [58], Shkurnikov et al. [56], Kirschner et al. [61], Pritchard et al. [62]
**20a**	no	MacLellan et al. [59], Shkurnikov et al. [56]
**448**	no	
21	yes	MacLellan et al. [59], Kirschner et al. [61], Pritchard et al. [62], Yamada et al. [60]
21-5p	yes	MacLellan et al. [59], Kirschner et al. [61], Pritchard et al. [62], Yamada et al. [60]
**145**	no	
141	no	
193b	no	
200b	no	
126	yes	MacLellan et al. [59], Kirschner et al. [61], Pritchard et al. [62]
301	no	MacLellan et al. [59]
328	no	MacLellan et al. [59], Pritchard et al. [62]
4478	no	
125b	no	Yamada et al. [60]
**1244**	no	
182	no	MacLellan et al. [59]
425-3p	no	MacLellan et al. [59]
**628-3p**	no	MacLellan et al. [59]
**29c**	no	MacLellan et al. [59]
429	no	
22	no	MacLellan et al. [59], Landoni et al. [58]
335-3p	no	
532	no	MacLellan et al. [59], Kirschner et al. [61]
**210**	no	MacLellan et al. [59], Kirschner et al. [61]
183	no	MacLellan et al. [59]
15b	yes	MacLellan et al. [59], Kirschner et al. [61], Pritchard et al. [62], Shah et al. [57], McDonald et al. [63]
92a	yes	MacLellan et al. [59], Landoni et al. [58], Kirschner et al. [61], Pritchard et al. [62]
17	yes	MacLellan et al. [59], Shkurnikov et al. [56], Kirschner et al. [61], Pritchard et al. [62]
103	yes	MacLellan et al. [59], Kirschner et al. [61], Pritchard et al. [62]
29a	yes	MacLellan et al. [59], Pritchard et al. [62], Yamada et al. [60]
24-3p	no	MacLellan et al. [59]

[a]miRNA affected by hemolysis as documented by three or more relevant studies.
[b]Studies documenting that given miRNA is affected by hemolysis (see text).
miRNAs in bold are those described in Table 2.
