# Supplementary material for: Systematic review and critique of circulating miRNAs as biomarkers of stage I-II non-small cell lung cancer
Source: Oncotarget. 2017 Oct 11;8(55):94980–96. doi: 10.18632/oncotarget.21739 (PMC5706930; doi:10.18632/oncotarget.21739)
Supplement: Supplementary file 2 [file oncotarget-08-94980-s002.docx]

**Supplementary File 1: Biological functions of microRNAs proposed as biomarkers for two-step screening of stage I-II NSCLC in the serum.**

**miR-223-3p**

Different effects of miR-223 have been shown in different types of cancer, and information about the role in lung cancer is limited. miR-223 behaves as a tumor suppressor in non-small cell lung cancer (NSCLC) and NSCLC cell line [1-3], a cervical cancer cell line and tumor tissues [4] and in acute myeloid leukemia [5, 6]. Conversely, miR-223 is overexpressed in gastric cancer compared to normal gastric mucosa and plays a role as an oncomiR in T-cell acute lymphoblastic leukemia [7] as well as in a gastric cancer cell line [8] and in prostate cancer cell lines [9]. The different behavior of miR-223 as oncogene or oncosuppressor could depend on its targets in the different tissues and cell types [10].

miR-223 inhibits cell growth, proliferation, colony formation in Hela cells in vitro and tumor formation in vivo by targeting insulin-like growth factor 1 (IGF-1R) and its downstream PI3K/Akt/mTOR/p70S6K pathway; this suppressive mechanism has also been confirmed in leukemia and hepatoma cells [11]; targeting of this pathway by re-expression of miR-223 also sensitizes erlotinib resistant lung cancer cells to drug-induced apoptosis [1].

miR-223 inhibits metastasis of cervical cancer by controlling epithelial-mesenchymal transition (EMT), upregulating the epithelial markers E-cadherin and α-cadherin and downregulating the mesenchymal marker vimentin [4]; conversely, inhibition of miR-223 in gemcitabine resistant pancreatic cancer cells reversed EMT [12].

In a human osteosarcoma cell line, miR-223 repressed the expression of a member of HSP90 family of stress induced proteins, inducing apoptosis as well as G0/G1 arrest [13].

*References*

*1. Zhao FY, Han J, Chen XW, Wang J, Wang XD, Sun JG, Chen ZT. miR-223 enhances the sensitivity of non-small cell lung cancer cells to erlotinib by targeting the insulin-like growth factor-1 receptor. Int J Mol Med. 2016; 38: 183-91. doi: 10.3892/ijmm.2016.2588.*

*2. Geng Q, Fan T, Zhang B, Wang W, Xu Y, Hu H. Five microRNAs in plasma as novel biomarkers for screening of early-stage non-small cell lung cancer. Respir Res. 2014; 15: 149. doi: 10.1186/s12931-014-0149-3.*

*3. Lv S, Xue J, Wu C, Wang L, Wu J, Xu S, Liang X, Lou J. Identification of A Panel of Serum microRNAs as Biomarkers for Early Detection of Lung Adenocarcinoma. J Cancer. 2017; 8: 48-56. doi: 10.7150/jca.16644.*

*4. Tang Y, Wang Y, Chen Q, Qiu N, Zhao Y, You X. MiR-223 inhibited cell metastasis of human cervical cancer by modulating epithelial-mesenchymal transition. Int J Clin Exp Pathol. 2015; 8: 11224-9. doi:*

*5. Fazi F, Racanicchi S, Zardo G, Starnes LM, Mancini M, Travaglini L, Diverio D, Ammatuna E, Cimino G, Lo-Coco F, Grignani F, Nervi C. Epigenetic silencing of the myelopoiesis regulator microRNA-223 by the AML1/ETO oncoprotein. Cancer Cell. 2007; 12: 457-66. doi: 10.1016/j.ccr.2007.09.020.*

*6. Pulikkan JA, Dengler V, Peramangalam PS, Peer Zada AA, Muller-Tidow C, Bohlander SK, Tenen DG, Behre G. Cell-cycle regulator E2F1 and microRNA-223 comprise an autoregulatory negative feedback loop in acute myeloid leukemia. Blood. 2010; 115: 1768-78. doi: 10.1182/blood-2009-08-240101.*

*7. Mavrakis KJ, Van Der Meulen J, Wolfe AL, Liu X, Mets E, Taghon T, Khan AA, Setty M, Rondou P, Vandenberghe P, Delabesse E, Benoit Y, Socci NB, et al. A cooperative microRNA-tumor suppressor gene network in acute T-cell lymphoblastic leukemia (T-ALL). Nat Genet. 2011; 43: 673-8. doi: 10.1038/ng.858.*

*8. Li J, Guo Y, Liang X, Sun M, Wang G, De W, Wu W. MicroRNA-223 functions as an oncogene in human gastric cancer by targeting FBXW7/hCdc4. J Cancer Res Clin Oncol. 2012; 138: 763-74. doi: 10.1007/s00432-012-1154-x.*

*9. Wei Y, Yang J, Yi L, Wang Y, Dong Z, Liu Z, Ou-yang S, Wu H, Zhong Z, Yin Z, Zhou K, Gao Y, Yan B, et al. MiR-223-3p targeting SEPT6 promotes the biological behavior of prostate cancer. Sci Rep. 2014; 4: 7546. doi: 10.1038/srep07546.*

*10. Zhang B, Pan X, Cobb GP, Anderson TA. microRNAs as oncogenes and tumor suppressors. Dev Biol. 2007; 302: 1-12. doi: 10.1016/j.ydbio.2006.08.028.*

*11. Jia CY, Li HH, Zhu XC, Dong YW, Fu D, Zhao QL, Wu W, Wu XZ. MiR-223 suppresses cell proliferation by targeting IGF-1R. PLoS One. 2011; 6: e27008. doi: 10.1371/journal.pone.0027008.*

*12. Ma J, Fang B, Zeng F, Ma C, Pang H, Cheng L, Shi Y, Wang H, Yin B, Xia J, Wang Z. Down-regulation of miR-223 reverses epithelial-mesenchymal transition in gemcitabine-resistant pancreatic cancer cells. Oncotarget. 2015; 6: 1740-9. doi: 10.18632/oncotarget.2714.*

*13. Li G, Cai M, Fu D, Chen K, Sun M, Cai Z, Cheng B. Heat shock protein 90B1 plays an oncogenic role and is a target of microRNA-223 in human osteosarcoma. Cell Physiol Biochem. 2012; 30: 1481-90. doi: 10.1159/000343336.*

**miR-20a-5p**

miR-20a plays an oncogenic role in NSCLC by targeting genes involved in several aspects of tumorigenesis. An inverse correlation was shown between expression of miR-20 and expression levels of ferroportin, thus increasing iron availability supporting cell proliferation in NSCLC cell lines [1]; another target of miR-20a, both *in vivo* and *in vitro* is transforming growth factor-β (TGF-β) type II receptor [2]. In colon cancer, miR-20a directly targets Smad4 3’UTR, promoting invasion and metastasis *in vitro* and *in vivo*by a colorectal cancer cell line [3].

In Hela cells c-Myc regulates miR-20a to target E2F1 transcription factor [4]. In osteosarcoma, a novel miRNA-target gene regulatory network was identified between miR-20a and CCND2 (G1/S-specific cyclin-D2) that may play a role during cell proliferation [5]. Finally, miR-20a expression may be affected by radiation therapy regimens during breast cancer treatment [6]. In turn, miR-20a regulates the expression of known target genes of the miR-17-92 cluster (to which this miRNA belongs), such as mitogen-activated protein kinase (MAPK), ErbB, p53, Wnt, transforming growth factor-β (TGF-β), mTOR signaling pathways and cell cycle [6].

*References*

*1. Babu KR, Muckenthaler MU. miR-20a regulates expression of the iron exporter ferroportin in lung cancer. J Mol Med (Berl). 2016; 94: 347-59. doi: 10.1007/s00109-015-1362-3.*

*2. Yang S, Cho YJ, Jin L, Yuan G, Datta A, Buckhaults P, Datta PK. An epigenetic auto-feedback loop regulates TGF-beta type II receptor expression and function in NSCLC. Oncotarget. 2015; 6: 33237-52. doi: 10.18632/oncotarget.4893.*

*3. Cheng D, Zhao S, Tang H, Zhang D, Sun H, Yu F, Jiang W, Yue B, Wang J, Zhang M, Yu Y, Liu X, Sun X, et al. MicroRNA-20a-5p promotes colorectal cancer invasion and metastasis by downregulating Smad4. Oncotarget. 2016; 7: 45199-213. doi: 10.18632/oncotarget.9900.*

*4. O'Donnell KA, Wentzel EA, Zeller KI, Dang CV, Mendell JT. c-Myc-regulated microRNAs modulate E2F1 expression. Nature. 2005; 435: 839-43. doi: 10.1038/nature03677.*

*5. He C, Gao H, Fan X, Wang M, Liu W, Huang W, Yang Y. Identification of a novel miRNA-target gene regulatory network in osteosarcoma by integrating transcriptome analysis. Int J Clin Exp Pathol. 2015; 8: 8348-57.*

*6. Leung CM, Chen TW, Li SC, Ho MR, Hu LY, Liu WS, Wu TT, Hsu PC, Chang HT, Tsai KW. MicroRNA expression profiles in human breast cancer cells after multifraction and single-dose radiation treatment. Oncol Rep. 2014; 31: 2147-56. doi: 10.3892/or.2014.3089.*

**miR-448**

miR-448 expression is often decreased in human cancer tissues and is considered a tumor suppressor in hepatocellular carcinoma [1], ovarian [2] and breast cancer [3]. This was recently confirmed also for lung squamocellular carcinoma, where it may regulate cell growth and metastasis, possibly by targeting DCLK1 [4]. Xu et al confirmed downregulation of this miRNA in NSCLC [5].

More information is available on the biological role of miR-448 in other cancers. Several direct targets of miR-448 have been identified in cell lines from various tumors, including the serine/threonine kinase ROCK2 in a hepatocellular carcinoma cell line, the CXCL12 chemokine in an ovarian cancer cell line and SATB1 (AT-rich sequence-binding protein 1), which enhances NF-κB activity and increases Twist1 expression, thereby leading to EMT in a breast cancer cell line [1, 2, 5, 6].

However, an oncogenic role of miR-448 has also been reported in gastric cancer where miR-448 was shown to suppress the expression of KDM2B that directly inhibits Myc [7].

These findings imply that miR-448 may play different and tissue-specific roles and behave differently in various tumor types.

*References*

*1. Zhu H, Zhou X, Ma C, Chang H, Li H, Liu F, Lu J. Low Expression of miR-448 Induces EMT and Promotes Invasion by Regulating ROCK2 in Hepatocellular Carcinoma. Cell Physiol Biochem. 2015; 36: 487-98. doi: 10.1159/000430114.*

*2. Lv Y, Lei Y, Hu Y, Ding W, Zhang C, Fang C. miR-448 negatively regulates ovarian cancer cell growth and metastasis by targeting CXCL12. Clin Transl Oncol. 2015; 17: 903-9. doi: 10.1007/s12094-015-1325-8.*

*3. Bamodu OA, Huang WC, Lee WH, Wu A, Wang LS, Hsiao M, Yeh CT, Chao TY. Aberrant KDM5B expression promotes aggressive breast cancer through MALAT1 overexpression and downregulation of hsa-miR-448. BMC Cancer. 2016; 16: 160. doi: 10.1186/s12885-016-2108-5.*

*4. Shan C, Fei F, Li F, Zhuang B, Zheng Y, Wan Y, Chen J. miR-448 is a novel prognostic factor of lung squamous cell carcinoma and regulates cells growth and metastasis by targeting DCLK1. Biomed Pharmacother. 2017; 89: 1227-34. doi: 10.1016/j.biopha.2017.02.017.*

*5. Xu C, Zheng Y, Lian D, Ye S, Yang J, Zeng Z. Analysis of microRNA expression profile identifies novel biomarkers for non-small cell lung cancer. Tumori. 2015; 101: 104-10. doi: 10.5301/tj.5000224.*

*6. Li QQ, Chen ZQ, Cao XX, Xu JD, Xu JW, Chen YY, Wang WJ, Chen Q, Tang F, Liu XP, Xu ZD. Involvement of NF-kappaB/miR-448 regulatory feedback loop in chemotherapy-induced epithelial-mesenchymal transition of breast cancer cells. Cell Death Differ. 2011; 18: 16-25. doi: 10.1038/cdd.2010.103.*

*7. Hong X, Xu Y, Qiu X, Zhu Y, Feng X, Ding Z, Zhang S, Zhong L, Zhuang Y, Su C, Hong X, Cai J. MiR-448 promotes glycolytic metabolism of gastric cancer by downregulating KDM2B. Oncotarget. 2016; 7: 22092-102. doi: 10.18632/oncotarget.8020.*

**miR-145-5p**

The tumor suppression function of miR-145 is recognized by the scientific community [1, 2]. The oncosuppressive functions of miR-145 have been described in lung cancer tissues [3] and in lung, breast, prostate and colorectal cancer cell lines [4-6].

The expression of miR-145-5p in NSCLC is significantly lower than that in healthy tissues, potentially leading to an increase in genes such as SMAD4, SMAD2, IRS1, FOXO1, ERBB4, NRAS, ACTB, and ACTG1 that are predicted targets in NSCLC [7]. In another study miR-145 was shown to negatively correlate with N-cadherin expression in lung adenocarcinoma tissues and, by doing so, to be able to suppress cell invasion and migration in lung adenocarcinoma cell lines [8].

miR-145 also shows antitumor activity in lung squamocellular carcinoma cell lines and downregulation of this microRNA enhances the expression of metadherin (MTDH), a downstream mediator of several signal pathways, such as PI3K/AKT, NF-κB, MAPK and Wnt/β-catenin [9].

Several independent studies suggested that miR-145 could control NSCLC cell migration and invasion. Hu et al. demonstrated that miR-145 represses TGF-β-induced EMT and invasion by targeting SMAD3 in NSCLC cells; these authors showed that SMAD3, an intracellular mediator in TGF-β signaling, affected the expression of EMT markers as E-cadherin and N-cadherin [10]. Moreover, targeting of FSCN1 could also be a mechanism by which miR-145 controls EMT transition [11].

Finally, miR-145 was shown to regulate other tumor-associated targets such as c-Myc or mucin 1 in NSCLC cells [12, 13].

*References*

*1. Takaoka Y, Shimizu Y, Hasegawa H, Ouchi Y, Qiao S, Nagahara M, Ichihara M, Lee JD, Adachi K, Hamaguchi M, Iwamoto T. Forced expression of miR-143 represses ERK5/c-Myc and p68/p72 signaling in concert with miR-145 in gut tumors of Apc(Min) mice. PLoS One. 2012; 7: e42137. doi: 10.1371/journal.pone.0042137.*

*2. Sachdeva M, Zhu S, Wu F, Wu H, Walia V, Kumar S, Elble R, Watabe K, Mo YY. p53 represses c-Myc through induction of the tumor suppressor miR-145. Proc Natl Acad Sci U S A. 2009; 106: 3207-12. doi: 10.1073/pnas.0808042106.*

*3. Lu Y, Govindan R, Wang L, Liu PY, Goodgame B, Wen W, Sezhiyan A, Pfeifer J, Li YF, Hua X, Wang Y, Yang P, You M. MicroRNA profiling and prediction of recurrence/relapse-free survival in stage I lung cancer. Carcinogenesis. 2012; 33: 1046-54. doi: 10.1093/carcin/bgs100.*

*4. Zhou CH, Yang SF, Li PQ. Human lung cancer cell line SPC-A1 contains cells with characteristics of cancer stem cells. Neoplasma. 2012; 59: 685-92. doi: 10.4149/neo_2012_087.*

*5. Wang Z, Zhang X, Yang Z, Du H, Wu Z, Gong J, Yan J, Zheng Q. MiR-145 regulates PAK4 via the MAPK pathway and exhibits an antitumor effect in human colon cells. Biochem Biophys Res Commun. 2012; 427: 444-9. doi: 10.1016/j.bbrc.2012.06.123.*

*6. Gotte M, Mohr C, Koo CY, Stock C, Vaske AK, Viola M, Ibrahim SA, Peddibhotla S, Teng YH, Low JY, Ebnet K, Kiesel L, Yip GW. miR-145-dependent targeting of junctional adhesion molecule A and modulation of fascin expression are associated with reduced breast cancer cell motility and invasiveness. Oncogene. 2010; 29: 6569-80. doi: 10.1038/onc.2010.386.*

*7. Gan TQ, Xie ZC, Tang RX, Zhang TT, Li DY, Li ZY, Chen G. Clinical value of miR-145-5p in NSCLC and potential molecular mechanism exploration: A retrospective study based on GEO, qRT-PCR, and TCGA data. Tumour Biol. 2017; 39: 1010428317691683. doi: 10.1177/1010428317691683.*

*8. Mo D, Yang D, Xiao X, Sun R, Huang L, Xu J. MiRNA-145 suppresses lung adenocarcinoma cell invasion and migration by targeting N-cadherin. Biotechnol Lett. 2017; 39: 701-10. doi: 10.1007/s10529-017-2290-9.*

*9. Mataki H, Seki N, Mizuno K, Nohata N, Kamikawaji K, Kumamoto T, Koshizuka K, Goto Y, Inoue H. Dual-strand tumor-suppressor microRNA-145 (miR-145-5p and miR-145-3p) coordinately targeted MTDH in lung squamous cell carcinoma. Oncotarget. 2016; 7: 72084-98. doi: 10.18632/oncotarget.12290.*

*10. Hu H, Xu Z, Li C, Xu C, Lei Z, Zhang HT, Zhao J. MiR-145 and miR-203 represses TGF-beta-induced epithelial-mesenchymal transition and invasion by inhibiting SMAD3 in non-small cell lung cancer cells. Lung Cancer. 2016; 97: 87-94. doi: 10.1016/j.lungcan.2016.04.017.*

*11. Zhang Y, Lin Q. MicroRNA-145 inhibits migration and invasion by down-regulating FSCN1 in lung cancer. Int J Clin Exp Med. 2015; 8: 8794-802. doi:*

*12. Chen Z, Zeng H, Guo Y, Liu P, Pan H, Deng A, Hu J. miRNA-145 inhibits non-small cell lung cancer cell proliferation by targeting c-Myc. J Exp Clin Cancer Res. 2010; 29: 151. doi: 10.1186/1756-9966-29-151.*

*13. Ye Z, Shen N, Weng Y, Li K, Hu L, Liao H, An J, Liu L, Lao S, Cai S. Low miR-145 silenced by DNA methylation promotes NSCLC cell proliferation, migration and invasion by targeting mucin 1. Cancer Biol Ther. 2015; 16: 1071-9. doi: 10.1080/15384047.2015.1046024.*

**miR-628-3p**

Little information is available about the role of miR-628-3p in lung cancer. Bioinformatic analysis identified ATRX (SWI/SNF family of chromatin remodeling proteins), SLC45A2 (transporter protein), and TNRC6B (Argonaute protein) as potential targets of miR-628-3p in NSCLC [1].

miR-628-3p dysregulation was described in some subtypes of gastric cancer and in neuroblastoma [2-4]. In the latter, this miRNA was found to negatively regulate *MYCN* gene expression [4], whereas in gastric cancer miR-628-3p appeared to regulate differentiation through as yet unknown mechanisms [2].

Another study showed that miR-628-3p is overexpressed in pancreatic cancer patient sera and tissues, compared to healthy controls [5].

*References*

*1. Wang Y, Zhao H, Gao X, Wei F, Zhang X, Su Y, Wang C, Li H, Ren X. Identification of a three-miRNA signature as a blood-borne diagnostic marker for early diagnosis of lung adenocarcinoma. Oncotarget. 2016; 7: 26070-86. doi: 10.18632/oncotarget.8429.*

*2. Gao S, Zhou F, Zhao C, Ma Z, Jia R, Liang S, Zhang M, Zhu X, Zhang P, Wang L, Su F, Zhao J, Liu G, et al. Gastric cardia adenocarcinoma microRNA profiling in Chinese patients. Tumour Biol. 2016; 37: 9411-22. doi: 10.1007/s13277-016-4824-5.*

*3. Li FQ, Xu B, Wu YJ, Yang ZL, Qian JJ. Differential microRNA expression in signet-ring cell carcinoma compared with tubular adenocarcinoma of human gastric cancer. Genet Mol Res. 2015; 14: 739-47. doi: 10.4238/2015.January.30.17.*

*4. Megiorni F, Colaiacovo M, Cialfi S, McDowell HP, Guffanti A, Camero S, Felsani A, Losty PD, Pizer B, Shukla R, Cappelli C, Ferrara E, Pizzuti A, et al. A sketch of known and novel MYCN-associated miRNA networks in neuroblastoma. Oncol Rep. 2017; 38: 3-20. doi: 10.3892/or.2017.5701.*

*5. Li A, Yu J, Kim H, Wolfgang CL, Canto MI, Hruban RH, Goggins M. MicroRNA array analysis finds elevated serum miR-1290 accurately distinguishes patients with low-stage pancreatic cancer from healthy and disease controls. Clin Cancer Res. 2013; 19: 3600-10. doi: 10.1158/1078-0432.CCR-12-3092.*

**miR-29c-3p**

miR-29c downregulation was associated with unfavorable prognosis in lung adenocarcinoma. miR-29c expression inhibited cell proliferation, migration and invasion in lung cancer cell lines and reduced the capability of tumor cells to promote HUVEC tube formation. VEGFA was shown to be a direct target of miR-29c [1].

Fabbri et al showed that miR-29c targets DNA methyltrasferases 3A and 3B in lung cancer cell lines and in NSCLC tissues, thus suppressing oncogenic DNA methylation [2]. Moreover, miR-29c targets Sp1 and reduces TGF-β-induced EMT, expression of epithelial markers such as TTF-1, while enhancing cell migration and invasion of two lung cancer cell lines [3].

Conversely, other studies suggested an oncogenic role for miR-29c. miR-29c level was significantly increased in sera and tissues of stage IA/B NSCLC patients compared to corresponding control sera and noncancerous tissues [4, 5].

*References*

*1. Liu L, Bi N, Wu L, Ding X, Men Y, Zhou W, Li L, Zhang W, Shi S, Song Y, Wang L. MicroRNA-29c functions as a tumor suppressor by targeting VEGFA in lung adenocarcinoma. Mol Cancer. 2017; 16: 50. doi: 10.1186/s12943-017-0620-0.*

*2. Fabbri M, Garzon R, Cimmino A, Liu Z, Zanesi N, Callegari E, Liu S, Alder H, Costinean S, Fernandez-Cymering C, Volinia S, Guler G, Morrison CD, et al. MicroRNA-29 family reverts aberrant methylation in lung cancer by targeting DNA methyltransferases 3A and 3B. Proc Natl Acad Sci U S A. 2007; 104: 15805-10. doi: 10.1073/pnas.0707628104.*

*3. Zhang HW, Wang EW, Li LX, Yi SH, Li LC, Xu FL, Wang DL, Wu YZ, Nian WQ. A regulatory loop involving miR-29c and Sp1 elevates the TGF-beta1 mediated epithelial-to-mesenchymal transition in lung cancer. Oncotarget. 2016; 7: 85905-16. doi: 10.18632/oncotarget.13137.*

*4. Heegaard NH, Schetter AJ, Welsh JA, Yoneda M, Bowman ED, Harris CC. Circulating micro-RNA expression profiles in early stage nonsmall cell lung cancer. Int J Cancer. 2012; 130: 1378-86. doi: 10.1002/ijc.26153.*

*5. Zhu W, He J, Chen D, Zhang B, Xu L, Ma H, Liu X, Zhang Y, Le H. Expression of miR-29c, miR-93, and miR-429 as potential biomarkers for detection of early stage non-small lung cancer. PLoS One. 2014; 9: e87780. doi: 10.1371/journal.pone.0087780.*

**miR-210-3p**

In lung cancer the role of this miRNA has been scarcely investigated. miR-210-3p was found overexpressed in NSCLC tissues at late stages, and targeted speciﬁc mitochondrial components, thus regulating cell death and survival and modulating HIF-1 activity [1]. The latter authors reported that miR-210 directly targets NDUFA4 (subunit of NADH dehydrogenase) and SDHD (succinate dehydrogenase complex subunit D) and induces mitochondrial dysfunction. Daugaard et al showed that increased expression of miR-210-3p was significantly associated with the presence of distant metastases [2]. However, miR-210-3p dysregulation may occur early in lung tumorigenesis, as it was found increased in plasma or serum of patients with early lung cancer [3-5].

More information is available about miR-210 functions in the context of other malignancies. miR-210 is induced by hypoxia and, in turn, it is able to increase HIF-1 transcriptional activity and expression of its target genes, VEGF and carbonic anhydrase 9, in glioblastoma specimens and cell lines [6].

In metastatic prostate cancer, overexpression of miR-210-3p positively correlates with serum PSA levels, Gleason grade and bone metastasis status in prostate cancer patients [7]. Expression of this miRNA in prostate cancer cells positively correlates with EMT transition, invasion and metastasis by targeting negative regulators of NF-κB signaling [7].

Moreover, miR-210-3p was shown to downregulate PICK1, a negative regulator of the TGF-β signaling pathway, necessary for metastasis to the bone [8].

Although the serum levels of miR-210-3p were found upregulated in clear cell renal cell carcinoma [9, 10], miR-210-3p depletion was shown to increase tumorigenesis in xenografts *in vivo* and to alter the morphology of renal cancer derived cell lines, indicating EMT, *in vitro*. TWIST1 was identified as a key target of miR-210-3p [11].

*References*

*1. Puissegur MP, Mazure NM, Bertero T, Pradelli L, Grosso S, Robbe-Sermesant K, Maurin T, Lebrigand K, Cardinaud B, Hofman V, Fourre S, Magnone V, Ricci JE, et al. miR-210 is overexpressed in late stages of lung cancer and mediates mitochondrial alterations associated with modulation of HIF-1 activity. Cell Death Differ. 2011; 18: 465-78. doi: 10.1038/cdd.2010.119.*

*2. Daugaard I, Veno MT, Yan Y, Kjeldsen TE, Lamy P, Hager H, Kjems J, Hansen LL. Small RNA sequencing reveals metastasis-related microRNAs in lung adenocarcinoma. Oncotarget. 2017; 8: 27047-61. doi: 10.18632/oncotarget.15968.*

*3. Shen J, Liu Z, Todd NW, Zhang H, Liao J, Yu L, Guarnera MA, Li R, Cai L, Zhan M, Jiang F. Diagnosis of lung cancer in individuals with solitary pulmonary nodules by plasma microRNA biomarkers. BMC Cancer. 2011; 11: 374. doi: 10.1186/1471-2407-11-374.*

*4. Shen J, Todd NW, Zhang H, Yu L, Lingxiao X, Mei Y, Guarnera M, Liao J, Chou A, Lu CL, Jiang Z, Fang H, Katz RL, et al. Plasma microRNAs as potential biomarkers for non-small-cell lung cancer. Lab Invest. 2011; 91: 579-87. doi: 10.1038/labinvest.2010.194.*

*5. Zhu W, Zhou K, Zha Y, Chen D, He J, Ma H, Liu X, Le H, Zhang Y. Diagnostic Value of Serum miR-182, miR-183, miR-210, and miR-126 Levels in Patients with Early-Stage Non-Small Cell Lung Cancer. PLoS One. 2016; 11: e0153046. doi: 10.1371/journal.pone.0153046.*

*6. Agrawal R, Pandey P, Jha P, Dwivedi V, Sarkar C, Kulshreshtha R. Hypoxic signature of microRNAs in glioblastoma: insights from small RNA deep sequencing. BMC Genomics. 2014; 15: 686. doi: 10.1186/1471-2164-15-686.*

*7. Ren D, Yang Q, Dai Y, Guo W, Du H, Song L, Peng X. Oncogenic miR-210-3p promotes prostate cancer cell EMT and bone metastasis via NF-kappaB signaling pathway. Mol Cancer. 2017; 16: 117. doi: 10.1186/s12943-017-0688-6.*

*8. Dai Y, Ren D, Yang Q, Cui Y, Guo W, Lai Y, Du H, Lin C, Li J, Song L, Peng X. The TGF-beta signalling negative regulator PICK1 represses prostate cancer metastasis to bone. Br J Cancer. 2017; 117:685–94.*

*9. Petrozza V, Pastore AL, Palleschi G, Tito C, Porta N, Ricci S, Marigliano C, Costantini M, Simone G, Di Carlo A, Gallucci M, Carbone A, Fazi F. Secreted miR-210-3p as non-invasive biomarker in clear cell renal cell carcinoma. Oncotarget. 2017; 8:69551-69558. doi:* [*10.18632/oncotarget.18449*](https://doi.org/10.18632/oncotarget.18449)*.*

*10. Petrozza V, Carbone A, Bellissimo T, Porta N, Palleschi G, Pastore AL, Di Carlo A, Della Rocca C, Fazi F. Oncogenic MicroRNAs Characterization in Clear Cell Renal Cell Carcinoma. Int J Mol Sci. 2015; 16: 29219-25. doi: 10.3390/ijms161226160.*

*11. Yoshino H, Yonemori M, Miyamoto K, Tatarano S, Kofuji S, Nohata N, Nakagawa M, Enokida H. microRNA-210-3p depletion by CRISPR/Cas9 promoted tumorigenesis through revival of TWIST1 in renal cell carcinoma. Oncotarget. 2017; 8: 20881-94. doi: 10.18632/oncotarget.14930.*

**miR-1244**

miR-1244 was found hypoexpressed in lung tumors and its reintroduction reduced the growth of lung cancer xenografts [1]. The same authors showed that miR-1244 targets myocyte enhancer factor 2D (MEF2D) in lung cancer cells [1].

Conversely, Wang et al. demonstrated higher levels of this miRNA in the sera of stage I-II NSCLC patients compared to patients with unidentified pulmonary nodules and healthy controls [2].

miR-1244 was found downregulated in A549 lung cancer cell line upon acquisition of resistance to cisplatin [3]. Overall survival times of cisplatin-treated NSCLC patients with high miR-1244 expression were higher than those of patients with low miR-1244 expression [4]. miR-1244 affected cisplatin-treated NSCLC via MEF2D expression [4].

*References*

*1. Zhang R, Zhang Y, Li H. miR-1244/Myocyte Enhancer Factor 2D Regulatory Loop Contributes to the Growth of Lung Carcinoma. DNA Cell Biol. 2015; 34: 692-700. doi: 10.1089/dna.2015.2915.*

*2. Wang W, Li W, Ding M, Yuan H, Yang J, Meng W, Jin E, Wang X, Ma S. Identification of miRNAs as non-invasive biomarkers for early diagnosis of lung cancers. Tumour Biol. 2016; 37:16287–93.*

*3. Li W, Wang W, Ding M, Zheng X, Ma S, Wang X. MiR-1244 sensitizes the resistance of non-small cell lung cancer A549 cell to cisplatin. Cancer Cell Int. 2016; 16: 30. doi: 10.1186/s12935-016-0305-6.*

*4. Li GJ, Zhao GQ, Yang JP, Zhou YC, Yang KY, Lei YJ, Huang YC. Effect of miR-1244 on cisplatin-treated non-small cell lung cancer via MEF2D expression. Oncol Rep. 2017; 37: 3475-83. doi: 10.3892/or.2017.5624.*
