# Supplementary material for: Systematic review and critique of circulating miRNAs as biomarkers of stage I-II non-small cell lung cancer
Source: Oncotarget. 2017 Oct 11;8(55):94980–96. doi: 10.18632/oncotarget.21739 (PMC5706930; doi:10.18632/oncotarget.21739)
Supplement: Supplementary file 3 [file oncotarget-08-94980-s003.docx]

**Supplementary File 2: Model proposed to estimate the overall sensitivity and specificity of 8 miRNAs used for a two-step screening of stage I-II NSCLC. Test 1 is performed with a panel of 4 highly sensitive miRNAs (A,B,C,D), Test 2 with a panel of 4 highly specific miRNAs (E,F,G,H) (Table 4).**

Step 1) Within each panel the combined sensitivity for Test 1 (T1se) and Test 2 (T2se) and the combined specificity for Test 1 (T1sp) and Test 2 (T2sp) are calculated in parallel using the following formulas [1-3]:

-Combined sensitivity Test 1= 1 - (1-Ase) x (1-Bse) x (1-Cse) x (1-Dse)

-Combined specificity Test 1= Asp x Bsp x Csp x Dsp

-Combined sensitivity Test 2= 1 - (1-Ese) x (1-Fse) x (1-Gse) x (1-Hse)

-Combined specificity Test 2= Esp x Fsp x Gsp x Hsp

Where A-Hse, refer to the individual sensitivity value of each considered miRNA; A-Hsp refer to the individual specificity value.

Step 2) The overall sensitivity and specificity of Tests 1 and 2 are calculated in series using the following formulas:

-Overall sensitivity = T1se x T2se

-Overall specificity = T1sp + T2sp – (T1sp x T2sp)

Note: a subject is classified positive to screening if both Test 1 and Test 2 are positive; Test 2 is run only if Test 1 is positive. For either test, a single significantly dysregulated miRNA is enough to consider the test as positive.

**References**

1. Weinstein S, Obuchowski NA, Lieber ML. Clinical evaluation of diagnostic tests. AJR Am J Roentgenol. 2005; 184: 14-9. doi: 10.2214/ajr.184.1.01840014.
2. Fletcher R, Fletcher S. Clinical Epidemiology. Philadelphia [u.a.]: Lippincott Williams & Wilkins; 2009.
3. Zhou XH, Obuchowski NA, McClish DK. Statistical Methods in Diagnostic Medicine. Wiley; New York; 2002.
