# Supplementary material for: Systematic review and critique of circulating miRNAs as biomarkers of stage I-II non-small cell lung cancer
Source: Oncotarget. 2017 Oct 11;8(55):94980–96. doi: 10.18632/oncotarget.21739 (PMC5706930; doi:10.18632/oncotarget.21739)
Supplement: Supplementary file 4 [file oncotarget-08-94980-s004.docx]

Supplementary Table 1B: Materials and procedures used for miRNA quantification in the 20 included studies

| **Reference** | **Year** | **Starting material** | **Study**  **Validation (yes/no)** | **RNA isolation/ Spike-in** | **Retrotrascription/ Spike-in** | **Quantification method** | **Normalization** | **Analysis of results** | **Assessment of hemolysis** |
| --- | --- | --- | --- | --- | --- | --- | --- | --- | --- |
| **Bianchi et al. [26]** | **2011** | Serum | Yes | Trizol-LS (Invitrogen) combined with mirVana miRNA Isolation Kit (Ambion)/NA^a^ | TaqMan MicroRNA Reverse Transcription Kit & Taqman multiplex RT assays (AB)/NA | TLDA^b^ microRNA signature panel (AB^c^) | geometric mean of miR-197, miR-19a, miR-19b, miR-146, miR-15b, miR- 24 | 2^-ΔΔCt^ | NA |
| **Foss et al. [27]** | **2011** | Serum-plasma | Yes | Phenol and guanidine thiocianate (75-200 μL serum/plasma)/cel-miR-39 | 2) GenoExplorer miRNA First-Strand cDNA Core kit (Genosensor corp.)/NA | 1) array (Genosensor corp.)(discovery set); 2) qPCR (validation set): Sybr green (Roche) & miR^d^ specific forward primer+universal reverse primer) | 1) PC-U6B, U6-337, 5S-rRNA, PC-HU5S;  2) RNU6 or cel-miR-39 separately | 1-(C_tGOI_-C_tREF_)/maxC^e^ (unclear the normalization on the 2 reference miRs) | NA |
| **Geng et al. [48]** | **2014** | Plasma | Yes | NA /NA | NA & specific reverse primers /NA | Relative qPCR [MyScript SYBR green PCR kit (Qiagen)] | miR-16 | 2^-ΔΔCt^ (where ΔCt= C_tREF_-C_tGOI_) | NA |
| **Halvorsen et al. [47]** | **2016** | Serum | Yes | miRCURY RNA isolation kit (Exiqon) /NA | 1) TaqMan MicroRNA Reverse Transcription Kit & Megaplex RT primer pool (AB); 2) MicroRNA reverse trascription kit & stem-loop primers (AB)/NA | 1) TLDA microRNA signature panel (AB); 2) Relative qPCR (TaqMan Universal PCR Master Mix, no UNG & Taqman small RNA assays (AB) | 1) global normalization method; 2) geometric mean of miR-220, miR-19b, sU6, chosen from 1) | 2^-ΔΔCt^ | NA |
| **Li et al. [43]** | **2015** | Plasma | No | miRNeasy Mini Kit (Qiagen)/cel-miR-39 | Taqman MicroRNA Reverse Trascription Kit (AB) & stem-loop primers (AB)/NA | Relative qPCR with Taqman Universal Master Mix II & Taqman primers-probes (AB) | cel-miR-39 | 2^-ΔΔCt^ | NA |
| **Ma et al. [28]** | **2013** | Plasma | No | mirVana PARIS RNA kit (Ambion) /NA | Taqman MicroRNA Reverse Trascription Kit (AB) & stem-loop primers (AB)/NA | Droplet digital PCR (Taqman primers-probes) | absolute quantification | copies/µl plasma | NA |
| **Nadal** **et al. [45]** | **2015** | Serum | Yes | mirVana PARIS RNA kit (Ambion) /NA | TaqMan MicroRNA Reverse Transcription Kit & Megaplex RT primer pool (AB); preamplification/NA | 1) Taqman Openarray Human microRNA panel (AB); 2) Relative qPCR (Taqman Master Mix & Taqman primers-probes (AB) | 1) average Ct of all miRNAs as loading control; 2) U6 snRNA | 2^-ΔΔCt^ | NA |
| **Powrozek et al. [46]** | **2016** | Plasma | No | miRNAeasy serum and plasma kit (Qiagen)/NA | Taqman MicroRNA Reverse Trascription Kit (AB) & stem-loop primers (AB)/NA | Relative qPCR  (Taqman Universal Master Mix II with UNG & Taqman primer-probes (AB) | U6 snRNA | ΔCt, 2^-ΔCt^, 2^-ΔΔCt^ | NA |
| **Sanfiorenzo et al. [29]** | **2013** | Plasma | No | miRNAeasy Mini Kit (Qiagen)/NA | NA /NA | Relative qPCR (NA) | Mean of miR-192-5p, and miR-16-5p | ΔΔCt [( C_tGOI_-C_tREF_)-global mean of relative expression of each miRNA] | Yes (miR-16-5p and miR-103a-3p; miR-16-5p used both as hemolysis indicator and as reference) |
| **Shen et al. [30]** | **2011** | Plasma | Yes | mirVana PARIS RNA kit (Ambion) /cel-miR-238 | Taqman MicroRNA Reverse Trascription Kit (AB) & stem-loop primers (AB)/ NA | Relative qPCR (NA) | miR-16 | 2^-ΔΔCt^ | NA |
| **Shi et al. [41]** | **2017** | Serum | No | miRNA extraction kit (Tiangen Biology co.) /NA | NA | NA | miR-103 | 2^-ΔΔCt^ | NA |
| **Sun et al. [55]** | **2016** | Serum | No | RNA extraction kit (Applied Biosystem) /NA | Taqman MicroRNA Reverse Trascription Kit (AB) & Taqman microRNA assay/NA | Relative qPCR (Taqman Universal Master Mix & Taqman microRNA assay) | miR-16 | 2^-ΔΔCt^ | NA |
| **Ulivi et al. [49]** | **2013** | Blood | No | Trizol reagent (Invitrogen)/NA | Taqman MicroRNA Reverse Transcription kit (AB) & Taqman microRNA assay/NA | Relative qPCR (Taqman microRNA assay) | RNU38B, RNU58A | 2^-ΔΔCt^ | NA |
| **Wang Y. et al. [50]** | **2016** | Plasma | Yes | mirVana PARIS kit (Ambion)/cel-miR-39 | 1) Taqman MicroRNA Reverse Trascription Kit (AB) & Megaplex RT primer pool (AB); preamplification (Megaplex preamp primers)(AB) 2) Taqman MicroRNA Reverse Transcription kit (AB) & Taqman microRNA assay /cel-miR-39 | 1) TLDA cards (AB) 2) Relative qPCR (Taqman Universal Master Mix & Taqman primer-probes (AB) | cel-miR-39 | 2^-ΔΔCt^ | NA |
| **Wang W. et al. [53]** | **2016** | Serum | No | miRNAeasy Serum/Plasma Kit (Qiagen)/ cel-miR-39 | MyScript reverse transcription kit (Qiagen) | 2) Relative qPCR | cel-miR-39 | 2^-ΔΔCt^ | NA |
| **Wozniak et al. [54]** | **2015** | Plasma | No | NucleoSpin miRNA Plasma kit (Macherey-Nagel) /Ath-miR-159a | TaqMan MicroRNA Reverse Transcription Kit & Taqman primers (AB) / NA | 1) TLDA cards (AB) |  | Quantile normalization | Yes (none of the miRNAs described in literature as influenced by hemolysis) |
| **Yuxia et al. [42]** | **2012** | Serum | No | no extraction (quantification from serum) /NA | Taqman MicroRNA Reverse Transcription kit (AB) & Taqman microRNA assay (AB)/NA | Relative qPCR (Taqman microRNA assay) | NA | 2^50-Ct^ | NA |
| **Zhang et al. [51]** | **2017** | Plasma | Yes | Trizol+mirVana PARIS kit (Ambion)/NA | Taqman MicroRNA Reverse Trascription Kit (Ambion Life Technologies) & stem&loop primers | Relative qPCR (Brilliant III Ultra-Fast SYBR-Green qPCR master mix kit (Ambion Life Technologies) | miR-16 | 2^-ΔΔCt^ | NA |
| **Zhu et al. [44]** | **2014** | Serum | No | mirVana PARIS kit (Ambion) OR miRNA isolation kit (AB) /NA | Taqman MicroRNA Reverse Transcription Kit (AB) & Taqman probes/NA | Relative qPCR (Taqman Universal Master Mix & Taqman primer-probes (AB) | U6 and U48 snRNA | 2^-ΔΔCt^ | NA |
| **Zhu et al. [52]** | **2016** | Serum | No | mirVana PARIS kit (Ambion)/NA | Taqman MicroRNA Reverse Trascription Kit Applied Biosystems & Taqman probes/NA | Relative qPCR (Taqman Universal Master Mix & Taqman primer-probes (AB) | U6 snRNA | 2^-ΔΔCt^ | NA |

^a^ NA: Not available

^b^ TLDA: Taqman Low Density Array

^c^ AB: Applied Biosystems

^d^ miR: miRNA

^e^ max C: number of maximum cycles
