# Supplementary material for: Systematic review and critique of circulating miRNAs as biomarkers of stage I-II non-small cell lung cancer
Source: Oncotarget. 2017 Oct 11;8(55):94980–96. doi: 10.18632/oncotarget.21739 (PMC5706930; doi:10.18632/oncotarget.21739)
Supplement: Supplementary file 5 [file oncotarget-08-94980-s005.docx]

Supplementary **Table 1A. Main characteristics of the 20 included studies~~.~~**

| **Reference** | **Year** | **Country** | **Ethnicity** | **Sample Size^a^** | | **Mean Age** | | **Smokers** | | **Comorbidity**  **(case and control selection criteria)** | | **N. of miRNAs Individual/Panel** | **miRNAs Examined** | **NSCLC**^d^ **Stage** |
| --- | --- | --- | --- | --- | --- | --- | --- | --- | --- | --- | --- | --- | --- | --- |
|  |  |  |  | **Pt**^b^ | **C**^c^ | **Pt** | **C** | **Pt** | **C** | **Pt** | **C** |  |  |  |
| **Bianchi et al. [26]** | **2011** | Italy | Caucasian | 22 | 30 | 60 | matched | All smokers or former smokers (>20 pack-years) | | No history of cancer disease (in the last 5 years) | Healthy controls enrolled as part of the prospective COSMOS project (Low dose-CT^e^ always negative during the FU) | 34, panel | Panel of 34 miRNAs* | I |
| **Foss et al. [27]** | **2011** | USA | Caucasian | 31 | 22 | 62^#^ | 62^#^ | Smokers: 96,8% | Smokers: 100% | NA^f^ | Patients hospitalized for non-neoplastic and non-respiratory conditions (in ophthalmology or orthopedic department) | 2, panel | miR-1254 miR-574-5p | I-II |
| **Geng et al. [48]** | **2014** | China | Asian | 126 | 60 | 62.7%<60y 37.3%>60y | 58.3%<60y 41.7%>60y | Smokers: 42.9% Former smokers: 27.8% Non-smokers: 29.3% | Smokers: 28.3% Former smokers: 18.3% Non-smokers: 53.4% | No other cancer disease | 3 control groups: 1. no history of pulmonary disease; 2. Non-cancerous pulmonary disease; 3. benign pulmonary nodules | 5, individual | miR-20a,  miR-223,  miR-21,  miR-155, miR-145 | I-II |
| **Halvorsen et al. [47]** | **2016** | Norway | Caucasian | 79 | 58 | 62,6 | 57,6 | Smokers: 87% Non-smokers: 2% Unknown: 11% | Smokers: 100% Non-smokers: 0% | NA | IELCA screening trial samples with no cancer at lung CT | 6, panel | miR-125b, miR-200b, miR-34b, miR-203, miR-205, miR-429 | I-II |
| **Li et al. [43]** | **2015** | China | Asian | 11 | 11 | 59^#^ | 55^#^ | NA | | NA | No tumor history | 1, individual | miR-486 | I- II |
| **Ma et al. [28]** | **2013** | USA | African American, Caucasian | 36 | 38 | 66,7 | 64,6 | Smokers: 49,3% (28 pack-years) | Smokers: 19,6% (13 pack-years) | NA | No tumor history | 2, individual and panel | miR-21-5p, miR-335-3p | I |
| **Nadal** **et al. [45]** | **2015** | USA | NA | 75 | 60 | 65,5^#^ | 60^#^ | Smokers: 94% Non-smokers: 56% Unknown: 0 (0%) | Smokers: 48% Non-smokers: 26% Unknown: 26% | NA | No tumor history (mainly COPD^g^, bronchiectasis, pneumonia) | 4, individual and panel | miR-141, miR-200b, miR-193b,  miR-301 | I |
| **Powrozek et al. [46]** | **2016** | Poland | Caucasian | 29 | 85 | 64^#^ | 57^#^ | Smokers: 89.5% Non-smokers: 10.5% | matched | NA | No respiratory disease symptoms and chest X ray negative for lung disorders | 2, individual and panel | miR448, miR4478, combination of both miRNA | I-II |
| **Sanfiorenzo et al. [29]** | **2013** | France | NA | 35 | 20 | 65,1 | 67,5 | Smokers: 85% | Smokers: 75% | NA (3 people died for cause other than NSCLC) | 2 control groups: 1.COPD patients with no lung cancer or other malignancies;  2. healthy volunteers matched for smoking status | 12, panel | Panel of 12 miRNAs** | I-II |
| **Shen et al. [30]** | **2011** | USA | African American, Caucasian | 30 | 29 | 67,4^#^ SCC^h^  68^#^ AC^i^ | 66^#^ | Smokers:100% (31-35 pack-years) | Smokers:100% (30 pack years) | NA | | 4, panel | miR-21, miR-126, miR-210, miR-486-5p | I-II |
| **Shi et al. [41]** | **2017** | China | NA | 46 | 45 | 50,1 | 39,4 | Smokers:46,7 Non-smokers: 56,4% | Smokers: 31,1% Non-smokers: 68,9% | NA | Without disease (absence of hypertension, high cholesterol, diabetes) | 3, individual | miR-22, miR-125b, miR-15b | I-II |
| **Sun et al. [55]** | **2016** | China | Asian | 22 | 60 | 66,9 | 62,4 | NA | | NA | | 1, individual | miR-21 | I-II |
| **Ulivi et al. [49]** | **2013** | Italy | Caucasian | 54 | 24 | 68^#^ | 65^#^ | Smokers: 25.6% Former smokers: 53.5%  Non-smokers: 12.8%  Unknown: 8.1% | Smokers: 25% Former smokers: 29.2%  Non-smokers: 45.8% | No history of previous diseases | No tumor history | 1, individual | miR-328 | I-II |
| **Wang Y. et al. [50]** | **2016** | China | Asian | 82 | 91 | 59,8 | 61,4 | Smokers: 55.9% | Smokers: 47.8% | Not taking antibiotics or steroid, no HIV, HCV, HBV, no chemotherapy/radiotherapy) | 4 control groups: 1. lung benign disease, 2. other subtypes of lung cancer; 3.other adenocarcinomas 4.healthy controls | 3, individual and panel | miR-532, miR-628, miR-425-3p | I-II |
| **Wang W. et al. [53]** | **2016** | China | NA | 54 | 15 | 60^#^ | 51^#^ | NA | | NA | | 1, individual | miR-1244 | I-II |
| **Wozniak et al. [54]** | **2015** | Russia | Caucasian | 70 | 100 | 62,6 | 60,1 | Smokers: 54% Former Smokers:27% Non-smokers:11%: | Smokers:53% Former S.:11% Non-smokers:36% | NA | Individuals visiting two Moscow general hospitals for disorders unrelated to lung cancer and with associated risk factors | 24, panel | Panel of 24 miRNAs*** | I-II |
| **Yuxia et al. [42]** | **2012** | China | Asian | 76 | 110 | 43.5%<60y 56.5%>60y | 51.8%<60y 48.2%>60y | NA | | NA | No tumor history | 1, individual | miR-125b | I-II |
| **Zhang et al. [51]** | **2017** | China | NA | 109 | 63 | 59,3 | 59,7 | Smokers: 58,7% Non-smokers: 41,3% | Smokers: 42,9% Non-smokers: 57,1% | No previous history of cancer-related disease; no radio- or chemo-therapy prior to surgery | Without tumor-associated lesions confirmed by chest CT, blood test and other full body examinations | 4, individual and panel | miR-145, miR-20a, miR-21,  miR-223 | I-II |
| **Zhu et al. [44]** | **2014** | China | Asian | 36 | 48 | 59 | matched | NA | | NA | | 3, individual | miR-29c,  miR-93,  miR-429 | I |
| **Zhu et al. [52]** | **2016** | China | Asian | 87 | 40 | 58,5 | 57,9 | Smokers:35.7% Non-smokers: 64.3% | All non-smokers. 20 smokers individual were also collected as comparison group. | No chemotherapy / radiotherapy, no antibiotic therapy; no COPD, no others chronic conditions | 4 control groups: 1. pneumonia; 2. gastric cancer; 3. healthy smokers; 4. healthy non-smokers | 4, individual and panel | miR-182, miR-183, miR-210, miR-126 | 0-I |

^a^The sample refers to stage I and II NSCLC

^b^Pt=Patients; ^c^C=Controls

^d^NSCLC: non-small cell lung cancer

^e^CT: computed tomography

^f^NA: Not available

^g^COPD: chronic obstructive pulmonary disease

^h^SCC=squamous cell carcinoma; ^i^AC=adenocarcinoma

^#^median age

*miR-92a, miR-484, miR-486-5p, miR-328, miR-191, miR-376a, miR-342-3p, miR-331-3p, miR-30c, miR-28-5p, miR-98, miR-17, miR-26b, miR-374a, miR-30b, miR-26a, miR-142-3p, miR-103, miR-126, let-7a, let-7d, let-7b, miR-32, miR-133b, miR-566

**miR-155-5p, miR-20a-5p, miR-25-3p, miR-296-5p, miR-191-5p, miR-126-3p, miR-223-3p, miR-152-3p, miR-145-5p, miR-199a-5p, miR-24-3p, and let-7f-5p

***let-7c, miR-122, miR182, miR193a-5p, miR200c, miR203, miR218, miR 155, let-7b, miR-411, miR450b-5p, miR-485-3p, miR519a, miR-642, miR-517b, miR-520f, miR206, miR-566, miR-661, miR-340, miR-1241, miR-720, miR-543, miR1267.
